# Cognitive Framework for Aircraft Piloting: A Core Cognition Set

**DOI:** 10.3390/bs16081348

**Published:** 2026-08-05

**Authors:** Hongyi Huang, Yizhen Guo, Junsong Lu, Fan Li, Yin Wu

**Affiliations:** 1 Department of Applied Social Sciences, The Hong Kong Polytechnic University, Hong Kong, China; 2 Department of Psychology, University of California San Diego, La Jolla, CA 92093, USA; 3 Department of Aeronautical and Aviation Engineering, The Hong Kong Polytechnic University, Hong Kong, China; 4 Mental Health Research Centre, The Hong Kong Polytechnic University, Hong Kong, China

**Keywords:** cognitive function, piloting, aviation, cognitive assessment, risk factor

## Abstract

Human factors remain the predominant contributors to aviation accidents, yet the cognitive foundations of pilot performance have not been systematically defined. Existing cognitive frameworks inadequately represent the complex, high-demand environment of flight operations. This systematic review and meta-analysis aimed to identify a core cognition set for piloting, defined as the minimal group of cognitive modules most consistently related to flight performance, and to examine how these modules are affected by aviation-specific risk factors. A total of 93 studies were included in this review. Of these, 31 reported quantitative associations between cognitive performance and flight outcomes. A three-level mixed-effects meta-regression model was applied to estimate pooled effect sizes for each cognitive module. Meanwhile, 74 studies examined the influence of aviation factors such as fatigue, hypoxia, gravitational load, and aging. Meta-analytic findings indicated four cognitive modules (Perception, Working Memory, Multitasking Flexibility, and Psychomotor) as showing the strongest and most reliable associations with flight performance (Fisher’s z = 0.356–0.440, *p* < 0.001). The narrative synthesis corroborated that these four modules were most sensitive to operational and physiological risks such as fatigue, sleep deprivation, hypoxia, and aging. Working memory and cognitive flexibility consistently emerged as the earliest indicators of cognitive deterioration.

## 1. Introduction

### 1.1. Background: Human Factors in Aviation Safety

Aviation safety has traditionally been approached through the lens of aircraft design, engineering reliability, and procedural standardization. Yet, decades of accident investigation have consistently indicated that the human element remains the most critical component in the aviation system. Human factors have been implicated in approximately 70% to 80% of aviation accidents across both civil and military aviation (Wiegmann & Shappell, 2003). A review conducted by the National Academies found that human factors contribute to approximately 70% of all accidents and incidents, with values ranging from 60% to 85% across different databases (National Research Council et al., 1998). Among all human factors, pilot error emerged as the dominant human error type, which is responsible for 45% of all accidents in general aviation (Yilmaz, 2025). The dominance of human factors reflects the fundamental nature of aviation as a human-centered enterprise. Despite remarkable technological progress in aircraft automation, navigation systems, and onboard diagnostics, the pilot remains the ultimate decision-maker, particularly during critical phases of flight such as takeoff and landing (Kelly & Efthymiou, 2019). Technical failures account for only about 10% to 20% of causative factors, underscoring that the greatest leverage for improving aviation safety lies in understanding and supporting the human operator (National Research Council et al., 1998).

It is within this safety-critical context that researchers have turned their attention to pilots’ psychological and cognitive states. Cognitive function, the set of mental processes that mediate human interaction with the external environment (Lezak et al., 2012), has emerged as a fundamental lens for evaluating pilots’ operational readiness, capabilities, and vulnerabilities (Martinussen & Hunter, 2017). From perceptual scanning of instrument panels to working memory for flight parameters, from psychomotor coordination during manual control to multitasking across communication, navigation, and aircraft management, cognitive processes underpin virtually every aspect of flight operations (Tsang, 1998; Kay, 1995). Understanding how these cognitive functions operate and how they degrade under stress, fatigue, or other aviation-specific risk factors is therefore not an academic exercise but a practical imperative for flight safety.

### 1.2. Research Gap: The Mismatch Between Generic Cognitive Frameworks and Aviation-Specific Needs

Despite the recognized importance of cognitive function in piloting, the field lacks a clearly delineated, aviation-specific set of cognitive modules that are demonstrably critical to flight operations. First, generic cognitive frameworks were designed to describe cognition in the general population or in clinical populations, not to capture the unique cognitive demands of high-stakes, time-pressured, multi-dimensional flight operations. Current operational tools, such as the abbreviated Psychomotor Vigilance Task (PVT; Wilkinson & Houghton, 1982) used in fatigue management systems (FAA, 2013; ICAO, 2020; IATA et al., 2015), engage only a narrow subset of cognitive functions—primarily sustained attention and simple reaction time—and thus may offer an unduly reductive characterization of pilots’ cognitive states. Second, general cognitive frameworks (e.g., the framework adopted in Su et al., 2024) often include cognitive dimensions that have little documented relevance to actual piloting (such as certain subtypes of memory or abstract reasoning) while potentially overlooking modules that are critical in the cockpit but less emphasized in general cognitive theory. Third, the predominance of theory-driven frameworks has created a disconnect between laboratory-based cognitive constructs and the real-world demands of flight. Existing pilot selection batteries have been criticized for predicting training performance rather than operational performance, with low correlations between predictors and criterion measures (ALMamari & Traynor, 2019; Damos, 1996). There is currently not a comprehensive understanding of the cognitive abilities and psychological attributes necessary not only for effective pilot performance but also for a successful career as an aviator.

In short, while the importance of cognitive function in aviation is widely acknowledged, the field continues to operate without an empirically grounded, operationally relevant framework that specifies which cognitive modules matter most for piloting and how they should be assessed.

### 1.3. Core Cognition Set for Piloting

In response to these limitations, the present review introduces the concept of a ‘core cognition set’ for piloting. By ‘core,’ we refer to a minimal set of cognitive modules that demonstrate the most consistent empirical associations with pilots’ flight performance across diverse operational contexts. This set is derived through a systematic, evidence-driven synthesis guided by two considerations.

First, empirical significance: the module must demonstrate consistent associations with flight performance outcomes, providing sufficient evidence that it captures cognitive processes essential to piloting. Second, theoretical scope: for modules with acceptable but not overwhelming empirical support, we further considered whether the module, by its functional definition, captures a sufficiently broad range of piloting-relevant cognitive processes, rather than a narrow, task-specific operation. This additional consideration helps ensure that the final set is composed of modules with generalizable relevance to piloting, avoiding an overly fragmented collection of narrow correlates.

### 1.4. The Present Review

The main goal of the current review is to form a flight-task-specific cognitive set. To achieve this goal, the present review adopts a bottom-up, data-driven inductive approach, systematically synthesizing empirical evidence accumulated over the past 30 years (1996–2024) and focusing exclusively on peer-reviewed studies that report quantifiable associations between objective cognitive test performance and flight performance outcomes. The inclusion of each cognitive module was guided by two criteria: the strength of its empirical association with flight performance, and its theoretical relevance to the cognitive demands of piloting.

After establishing this cognition set, we conduct a narrative synthesis of the literature to determine whether these modules are also sensitive to key aviation-related risk factors such as fatigue, hypoxia, +Gz exposure, and aging. This secondary analysis is not used as a selection criterion, but rather as supplementary validation, providing evidence that the identified modules are not only associated with performance under optimal conditions but also susceptible to degradation under conditions that pose risks to flight safety.

## 2. Method

To identify and synthesize relevant evidence, this review conducted a systematic review following the guidelines of Preferred Reporting Items for Systematic reviews and Meta-Analyses (PRISMA).

### 2.1. Search Strategy

Articles for this review were identified in three ways. First, Web of Science, PubMed, and Scopus databases were searched for literature from the earliest possible start date until 24 February 2025. The literature collections for each database can be accessed via the corresponding .bib files on the OSF repository (URL: https://osf.io/6kw5j, accessed on 2 August 2026). Full texts were searched using the following terms. The specific Boolean operators applied in each database are detailed in Table S1.

((aviation) OR (flight) OR (aircraft)) AND ((pilot) OR (pilots)OR (aviator)) AND ((cognition) OR (cognitive) OR (working memory) OR (language) OR (executive function) OR (executive functions) OR (memory) OR (problem-solving) OR (mental rotation) OR (perception) OR (psychomotor) OR (Neuropsychological)).

### 2.2. Inclusion and Exclusion Criteria

Articles were included or excluded based on the following seven criteria: (1) investigated pilots’ cognitive functions and related human factors in aviation operations; (2) full-text English version of the article could be accessed and reviewed; (3) article reported original empirical data, including experimental and quasi-experimental designs involving behavioral data collection; (4) article provided sufficient information on experimental design, implementation procedures, and data collection methods to allow interpretation and replication; (5) article provided quantitative behavioral measures reflecting cognitive processes, based on cognitive paradigms or their modified forms; (6) sample consisted of licensed pilots (private pilot, commercial pilot, air force pilot, airline transport pilot, flight instructor) or recognized pilot cadets currently undergoing flight training; and (7) article reported quantitative results of cognitive assessments on pilot participants, not merely qualitative or diagnostic descriptions. More details referring to screening and data extraction could be retrieved in the Supplementary Document Table S2.

### 2.3. Cognitive Module Classification

Prior to data extraction, no fixed set of cognitive modules was adopted. Instead, we first recorded the specific cognitive paradigms or behavioral tasks, adopted as instruments that objectively assessed internal cognitive processes through quantifiable behavioral responses such as reaction time and accuracy. The original interpretations of measurement outcomes in each article were documented, though these were treated as references only. Because identical paradigms were labeled differently across studies. For example, the Psychomotor Vigilance Task (PVT) has been variously interpreted as a measure of attention, vigilance, or processing speed; similarly, the Wisconsin Card Sorting Test (WCST) has been described as indexing cognitive flexibility or executive function. To maintain consistency, two reviewers independently re-classified each paradigm based on a four-stage procedure, with theory-driven and data-driven decisions:

Stage 1 (theory-driven initial module classification). Two reviewers first constructed a preliminary classification framework by drawing on multiple widely used cognitive taxonomies and terms (Lezak et al., 2012; Su et al., 2024; Harvey, 2019; Johnson et al., 2017). Within this framework, some modules were operationally and conceptually indistinguishable (e.g., attention and processing speed, both relying on stimulus detection and rapid response), and thus were merged. All paradigms with unambiguous cognitive mappings were directly assigned to their corresponding module at this stage. For paradigms whose construct interpretations varied across the included studies, the classification was informed by highly influential literature in cognitive psychology. For instance, PVT was assigned to the attention module based on the definition of Lim and Dinges (2008). Based on Baggetta and Alexander (2016), who conceptualised executive function as a composite construct encompassing working memory, inhibition, and flexibility, WCST was not treated as a standalone indicator of the entire executive function domain. Instead, it was assigned specifically to the flexibility module, consistent with the classification of Miles et al. (2021).

In addition, certain modules (such as mental arithmetic, multitasking, and searching) engage multiple cognitive processes rather than mapping onto a single latent construct. Paradigms falling into these ambiguous modules were therefore carried forward for further adjudication in Stage 3.

Stage 2 (data-driven pruning). The preliminary module classification framework was refined by excluding any potential module for which no corresponding cognitive paradigm appeared in the included studies. For example, although social cognition (e.g., emotion recognition) is a well-established domain, no retrieved study employed such tasks; consequently, this candidate module was not retained for subsequent steps. After this pruning process, the framework retained nine modules: perception, working memory, flexibility, inhibition, psychomotor, long-term memory, problem-solving, spatial representation and language.

Stage 3 (theory-driven mapping of composite paradigms). Paradigms falling into these ambiguous modules in Stage 1 were assigned to specific modules using external authoritative reference. Three reclassification decisions were central to this stage: (1) Mental arithmetic was placed into *working memory*, as the Arithmetic subtest is a core component of the Working Memory Index in the Wechsler Adult Intelligence Scale–Fourth Edition (WAIS-IV; Wechsler, 2008), and empirical evidence further supports its strong association with working memory (Raghubar et al., 2010). (2) For visual searching and matching tasks (e.g., Trail Making Test-A, Symbol Digit Coding), the present review followed the taxonomy of Su et al. (2024), whose *perceptual processing* category encompasses simple reaction, matching, and search tasks. Based on their category, the original attention module was broadened to include detection and discrimination within a fixed sensory field, and subsequently renamed the *perception* module. (3) Regarding multitasking paradigms, they were assigned to *cognitive flexibility* rather than treated as a separate module. Multitasking inherently requires the coordination of multiple processes, including information maintenance, task-set shielding, task-s shifting, and interference resolution. Given that cognitive flexibility is considered the foundational ability that enables adaptation to changing task demands and serves as a “bridge” for multiple processes (Koch et al., 2018), multitasking paradigms were assigned to the *cognitive flexibility* module.

Stage 4 (data-driven refinement). Final adjustments were made based on extracted data and statistical considerations. The *language* module (e.g., verbal fluency) was excluded, because although such tasks appeared in the literature, only one study (Morrow et al., 2003) reported an effect size linking language to flight performance. A single effect size precludes estimation of within-module heterogeneity, yields statistically unstable estimates with low power, and risks misleading conclusions. Consistent with the criterion that at least three effect sizes are required for a meaningful subgroup analysis (Kolovos et al., 2016), this module was excluded from further quantitative synthesis. Furthermore, although multitasking was conceptually assigned to cognitive flexibility, the extracted data revealed divergent trends between multitasking and rule-switching tasks. A comparison of the merged and split models favored the split specification (likelihood-ratio test: χ^2^(1) = 9.98, *p* = 0.0016; ΔAICc = −7.36; model comparison information is listed in Table S3; summary of merged model is shown in Table S4), which we retained in the main analysis. A similar pattern emerged within problem-solving, but further subdivision would have reduced the number of effect sizes below the three-study threshold (Kolovos et al., 2016); therefore, the single module was retained in the main analysis.

The final cognitive module framework was established to include eight domains: perception, working memory, flexibility (including multitasking and rule-switching), inhibition, psychomotor, long-term memory, problem-solving, and spatial representation. The definitions of all final modules and the complete list of paradigms subsumed under each are presented in Table S5.

### 2.4. Data Extraction

For each included study, the following information was extracted: (1) study characteristics (authors, year, and experimental design); (2) participant characteristics (sample size, age, and pilot type or license); (3) cognitive assessment details (module classification, paradigm name, and outcome metrics); (4) risk factor characteristics (type, manipulation or exposure protocol, and conditional parameters where applicable); and (5) key statistical results, including effect sizes, confidence intervals, *p*-values, and test statistics as originally reported. Data extraction was performed independently by two reviewers, with disagreements resolved through discussion. The extracted data are available in the OSF repository (URL https://osf.io/6kw5j, accessed on 2 August 2026, file: PRISMA_Data_Extraction.xlsx, sheet: “Data_Form”).

### 2.5. Quality and Bias Assessment

Study quality was assessed using the Joanna Briggs Institute (JBI) Critical Appraisal Checklists, with the specific checklist selected according to each study’s design, including the checklists for randomized controlled trials, quasi-experimental studies, cross-sectional studies, and cohort studies (Grammatopoulos et al., 2023). Two reviewers independently rated each applicable item as “YES,” “NO,” “UNCLEAR,” or “NOT APPLICABLE.” Complete item-level ratings are provided in the Supplementary Document (Table S6); raw data are available in the OSF (URL https://osf.io/6kw5j, accessed on 2 August 2026, file: PRISMA_Data_Extraction.xlsx, sheet: “Data_Form”). Inter-rater agreement was 96% across item-level ratings (Cohen’s *κ* = 0.91, 95% CI [0.87, 0.95]). Discrepancies were resolved by consensus, and the finalized ratings were used in all analyses.

For each study, a quality score was calculated as the proportion of criteria met among applicable criteria, using the formula: (number of “YES” items + 0.5 × number of “UNCLEAR” items)/(total applicable items). “UNCLEAR” items were weighted 0.5 to reflect their partial informational value relative to clear “YES” ratings. Studies scoring ≥ 75% were classified as high quality, 50–75% as moderate quality, and <50% as low quality. These thresholds were adopted to enable standardized synthesis across JBI checklists with different item counts: 13 for RCTs, 11 for cohort studies, 9 for quasi-experimental studies, and 8 for cross-sectional studies. Following the classification framework of Jemal et al. (2026), the 75% threshold was derived from 6.5/9 ≈ 72% and rounded up to 75%, whereas the 50% threshold was derived from 3.5/9 ≈ 39% and rounded up to a more conservative 50% cut-off. The original item-level appraisal data are available in the OSF repository (URL https://osf.io/6kw5j, accessed on 2 August 2026, file: PRISMA_Data_Extraction.xlsx, sheet: “Data_Form”).

Bias assessment was derived from the same item-level ratings, with item-to-domain mapping following the JBI Manual for Evidence Synthesis (Section 4.2.7). Six bias domains were evaluated: selection bias, performance bias, detection bias, attrition bias, confounding bias, and reporting bias. Notably, the specific domains assessed varied across study designs, as each JBI checklist comprised items tailored to its respective design type. For example, confounding bias is systematically evaluated in cross-sectional studies, but is less prominently featured in quasi-experimental checklists. Risk of bias scores were calculated using the same proportional formula applied to the items within each domain, with ≥75% classified as low risk, 50–75% as moderate risk, and <50% as high risk. The complete item-to-domain mapping for each JBI checklist is provided in Supplementary Table S4.

### 2.6. Meta-Analytic Procedure

#### 2.6.1. Effect Size Calculation

For each study reporting associations between a cognitive module and flight performance, the reviewer extracted or computed Fisher’s *z* as the effect size metric (Fisher, 1915). Positive effect sizes were oriented such that higher values indicate that better cognitive performance is associated with superior flight performance; effect sizes from different studies were adjusted to maintain a consistent interpretation direction.

For studies reporting results from analysis of variance (ANOVA) or *t*-tests, we converted *F*, *t*, or *η*^2^ statistics to *r* using standard formulas (Cohen, 1988; Rosenthal, 1994), and then applied Fisher’s *z* transformation. Where necessary, degrees of freedom or sample sizes were used to compute the conversion. For studies that reported significance without providing sufficient statistics for effect size computation, we adopted a conservative imputation strategy: (a) when a result was reported as non-significant without further details, we assigned *r* = 0 and *p* = 1.00; (b) when a result was reported as significant without further details, we assigned *r* = 0.10 and *p* = 0.05.

When studies reported outcomes through multiple paradigms within the same cognitive module, we extracted all eligible effect sizes to preserve maximal information, accounting for their statistical dependency through a three-level modeling approach (see Section 2.6.2). The standard error (SE) for each Fisher’s *z* was computed as $SE=1/\sqrt{n-3}$, where *n* is the sample size from which the effect size was derived.

#### 2.6.2. Three-Level Mixed-Effects Model

All meta-analyses were conducted using the *metafor* package (Viechtbauer, 2010) in R (Version 4.4.1) statistical software (R Core Team, 2024). Because the primary studies reported multiple effect sizes (e.g., correlations between multiple cognitive modules and flight performance within the same sample), the assumption of independent effect sizes was violated. To account for this dependency, we employed a three-level mixed-effects meta-regression model (Cheung, 2014; Assink & Wibbelink, 2016). This approach partitions the total variance of effect sizes into three distinct levels: (a) sampling variance of the individual effect sizes (Level 1), (b) variance between effect sizes nested within the same study (Level 2), and (c) variance between studies (Level 3).

All effect sizes reflecting the associations between cognitive modules and flight performance were converted to Fisher’s *z* values prior to model fitting. The three-level model was specified with cognitive module categories as fixed-effect moderators, allowing for the direct estimation of the average effect size for each cognitive module without setting a reference category. The model was estimated using restricted maximum likelihood (REML).

#### 2.6.3. Heterogeneity Assessment

To assess the presence and magnitude of heterogeneity, we examined the variance components (i.e., τ^2^ at each level) and conducted a *Q*-test for residual heterogeneity. The proportion of total variance attributable to between-study and within-study heterogeneity was evaluated using the *I*^2^ statistic, calculated for each level following the approach described by Cheung (2014). In addition, the overall significance of the moderators was tested using the omnibus $Q_{M}$ statistic (Viechtbauer, 2010; Cheung, 2014).

#### 2.6.4. Sensitivity and Exploratory Moderator Analyses

To evaluate the stability of the findings, we conducted a leave-one-out sensitivity analysis, in which each study was sequentially removed, and the pooled effect size was recalculated (Viechtbauer & Cheung, 2010). Minimal variation across iterations indicated the robustness of the overall results.

To examine whether the results for cognitive modules were influenced by the type of flight-measure type, two additional models were fitted. First, an additive model included both cognitive module and flight-measure type as moderators. This model was treated as a sensitivity analysis assessing whether adjustment for flight-measure type improved model fit or altered the primary findings. Second, an exploratory interaction model included the interaction between cognitive module and flight-measure type to examine whether associations differed across flight-performance outcomes.

Three nested models were therefore compared: (1) the main cognitive-module model, (2) the additive model including cognitive module and flight-measure type, and (3) the interaction model including cognitive module, flight-measure type, and their interaction. Because the models differed in their fixed-effects structures, likelihood-ratio tests and information-criteria comparisons were conducted using maximum-likelihood estimation. Final parameter estimates were obtained using restricted maximum-likelihood estimation.

Flight-measure types were classified into three categories according to their measurement content: (1) composite performance type consisted of aggregate indices integrating multiple flight-task components (e.g., communication, traffic avoidance, emergency detection, and visual-approach performance in Adamson et al., 2010b), or overall flight-test evaluations provided by instructors (Leino et al., 1999); (2) flight control type consisted of objective, continuous indicators of aircraft-handling precision, including deviations in flight path, airspeed, altitude, and related flight parameters; and (3) situation awareness type comprised the measures assessing situation awareness (Xie et al., 2024), decision-making indicators (e.g., crosswind-landing decisions), and communication-based indicators (e.g., interactions with air traffic control in Morrow et al., 2003). Each effect size was assigned to one of these categories based on the outcome measure reported in the primary study. The complete coding scheme and type assignments are provided in the OSF repository (URL https://osf.io/6kw5j, accessed on 2 August 2026, file: PRISMA_Data_Extraction.xlsx, sheet: “Data_Form”).

#### 2.6.5. Publication Bias Assessment

Publication bias and small-study effects were examined using funnel plots, Egger’s regression test (Egger et al., 1997), and Duval and Tweedie’s trim-and-fill method (Duval & Tweedie, 2000). Following the recommendations of Stanley and Doucouliagos (2014), the Precision-Effect Test (PET) was also applied. Additionally, Rosenthal’s (1979) fail-safe *n* was computed to estimate the number of unpublished null studies required to eliminate the observed significance. These complementary tests provided a comprehensive evaluation of potential publication bias.

## 3. Results

### 3.1. Study Selection

Literature searches were conducted across Web of Science, PubMed, and Scopus on 24 February 2025, covering publications from 1 January 1995 to 24 February 2025. All retrieved records were exported in BibTeX (.bib) format and managed using Zotero (Version 7.0.9).

Batch deduplication was performed with the Zotero plugin Zoplicate (Version 3.0.8), followed by manual verification. After removing duplicates (*n* = 1962), books and book sections (*n* = 16), and irrelevant conference papers (*n* = 747), a total of 5357 unique records remained for screening. The complete reference library .bib file is publicly available on OSF (file: Reference_Library/references_dataset.bib).

Primary screening was independently performed by two reviewers based on titles, abstracts, and methods sections, using the predefined inclusion and exclusion criteria. Disagreements between reviewers were resolved through discussion until consensus was reached. During this stage, 5185 studies were excluded for reasons such as topic irrelevance, lack of accessible full text in English, or non-empirical study design. A total of 172 articles proceeded to the full-text eligibility stage.

Full-text screening was again performed independently by both reviewers, following the predefined criteria. Studies reporting duplicate data from identical samples and identical cognitive assessments were excluded, whereas studies reporting the same sample but different assessment paradigms or different statistical outcomes were retained. After resolving discrepancies through consensus discussion, 54 studies were excluded, resulting in 118 eligible studies included for data extraction.

**Figure 1:**
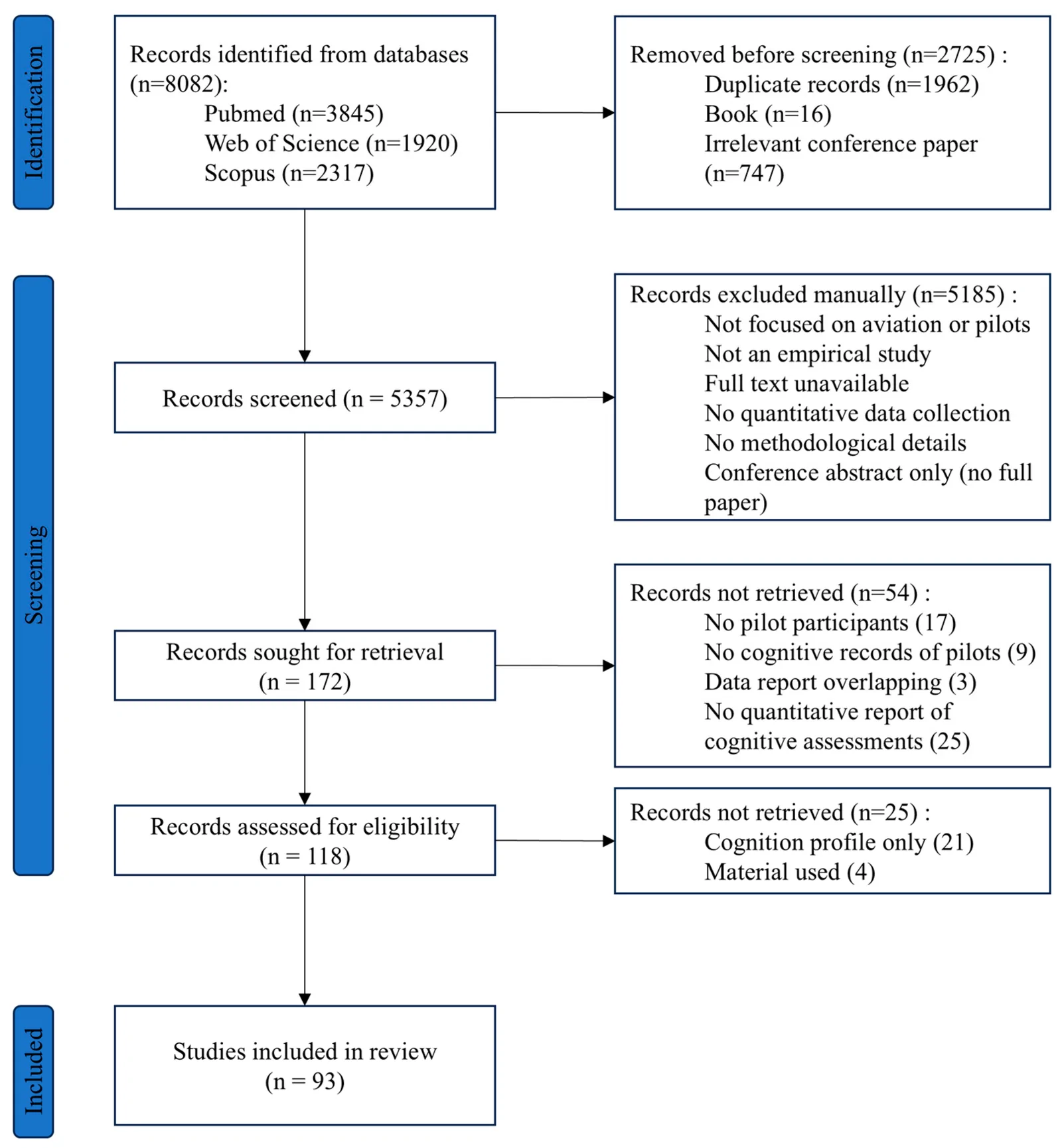
PRISMA Flow Diagram of Current Review. Note. The diagram illustrates the stages of the PRISMA workflow, including literature search, screening, and data extraction. Further methodological details and data extraction results can be found in the Supplementary Document.

Due to the subsequent narrowing of this review’s analytical scope, only 93 studies were ultimately included in the present synthesis and discussion. The complete selection process is summarized in the PRISMA flow diagram (Figure 1). The complete reference library .bib file is publicly available on OSF (file: Reference_Library/included_studies.bib).

### 3.2. Study Characteristics

Among 93 included studies, 31 studies reported quantitative associations between at least one cognitive module and flight performance, providing sufficient statistical information (e.g., correlations, *t*- or *F*-statistics, or standardized effect sizes) to be included in the three-level meta-analytic model. The list of included studies and corresponding information is presented in Table S10.

In addition, 74 studies investigated the effects of various aviation-related risk factors (e.g., fatigue, hypoxia, workload, stress, and sleep deprivation) on pilots’ cognitive modules. The list of included studies and corresponding information is presented in Table S10. These data were recorded but not synthesized quantitatively because of substantial methodological and conceptual heterogeneity across study designs. Findings from these studies were integrated into the Section 4, organized by the type of risk factor.

### 3.3. Meta-Analytic Estimates by Cognitive Module

Using a three-level mixed-effects meta-regression model to account for dependent effect sizes nested within studies, a total of 87 effect sizes from 31 studies were included. All effect sizes were expressed as Fisher’s *z* and estimated using restricted maximum likelihood (REML). The overall model, containing cognitive modules as fixed moderators, showed a significant overall association between pilots’ cognitive performance and flight performance ($Q_{M}$(9) = 187.12, *p* < 0.001).

Significant residual heterogeneity remained after accounting for cognitive modules ($Q_{E}$(78) = 156.75, *p* < 0.001). Variance components indicated small-to-moderate random variation at both the study (τ^2^study = 0.0089) and within-study levels (τ^2^within-study) = 0.0072). The proportion of total variance not attributable to sampling error was ${I^{2}}_{study}$ = 16.6%, ${I^{2}}_{within-study}$ = 13.4%, yielding a total *I*^2^ of approximately 29.9%. This suggests that roughly one-third of the observed variability in effect sizes reflects true differences rather than sampling error. As shown in Table S8, the main model significantly improved fit relative to the null model, χ^2^(8) = 25.75, *p* = 0.0012, and yielded a lower AICc. This comparison showed that cognitive modules explained 21.1% of the total variance (*R*^2^ = 0.211), driven primarily by a 53.2% reduction in between-study variance (τ^2^study: 0.0154 for null model; 0.0072 for main model), while within-study variance slightly increased (τ^2^within-study: 0.0050 for null model; 0.0089 for main model).

Meta-analytic estimates for each cognitive module are presented in Table 1. All cognitive modules demonstrated positive associations between pilots’ cognitive performance and flight performance. Among them, multitasking flexibility (*z* = 0.44, SE = 0.06, *p* < 0.001), perception (*z* = 0.43, SE = 0.04, *p* < 0.001), and working memory (*z* = 0.42, SE = 0.05, *p* < 0.001) yielded the largest effects, corresponding to approximately *r* ≈ 0.41–0.42 after back-transformation. Moderate positive effects were observed for psychomotor (*z* = 0.36, SE = 0.06, *p* < 0.001), spatial representation (*z* = 0.34, SE = 0.07, *p* < 0.001), and problem-solving (*z* = 0.35, SE = 0.14, *p* = 0.010). Smaller but statistically significant associations were found for long-term memory (*z* = 0.25, SE = 0.06, *p* < 0.001) and inhibition (*z* = 0.16, SE = 0.08, *p* = 0.046). By contrast, rule-switching flexibility showed the weakest yet statistically significant relationship (*z* = 0.20, SE = 0.06, *p* = 0.0003). A meta-analytic summary of the associations between pilots’ cognitive modules and flight performance is presented in Table 1, and detailed forest plots for each individual cognitive module are provided in the Supplementary Materials (Figures S1–S9). Each forest plot presents Fisher’s *z*-transformed correlation coefficients between pilots’ cognitive performance and flight performance across studies, along with corresponding 95% confidence intervals.

Overall, these results indicate that better cognitive performance is reliably associated with superior flight performance, particularly in domains involving complex coordination and attentional control (e.g., working memory and perception). The significant $Q_{M}$ test supports meaningful differences in effect strength among the cognitive modules, warranting further examination in subsequent analyses and discussion.

### 3.4. Study Quality and Risk of Bias

**Figure 2:**
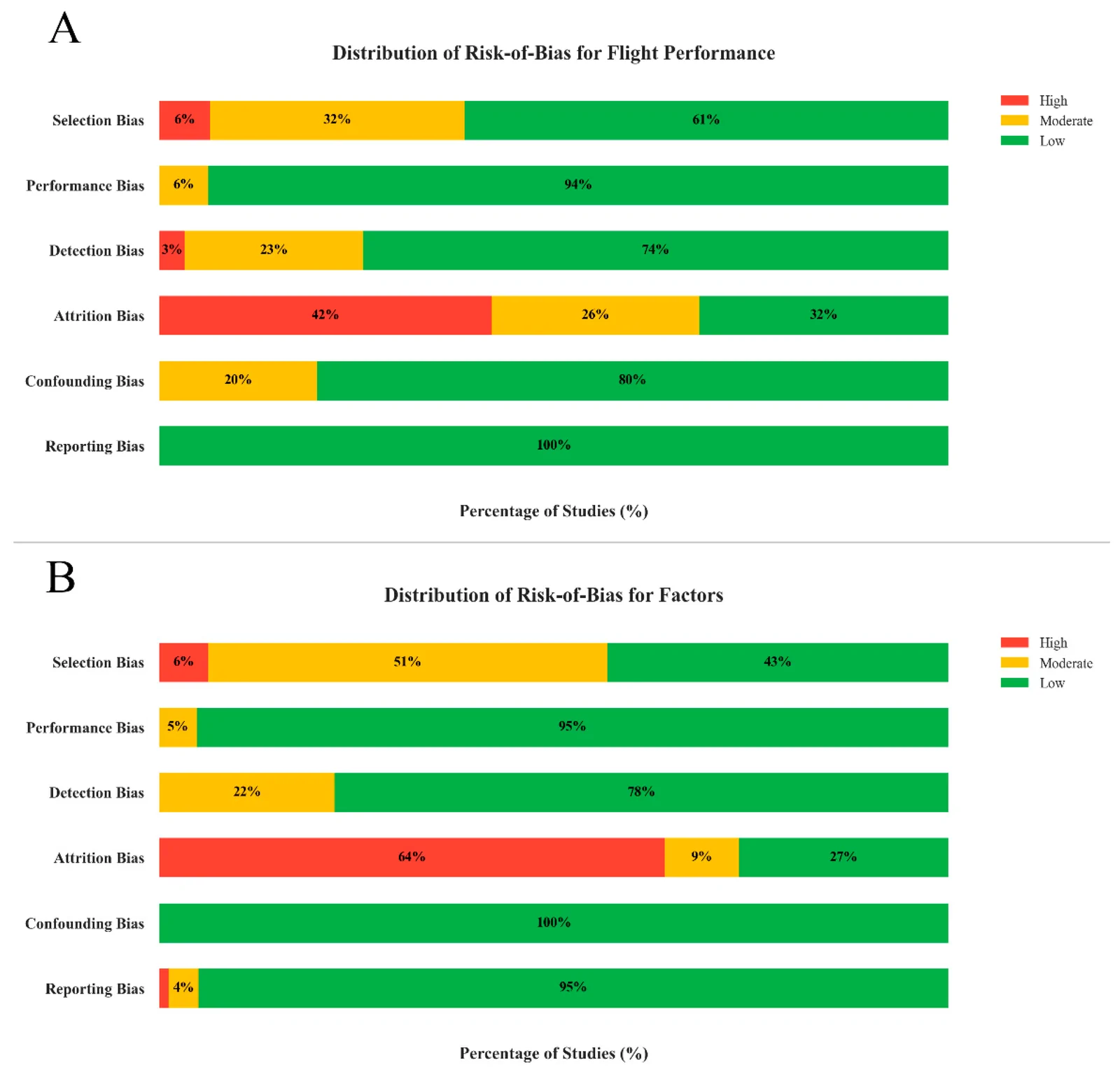
Summary of Risk-of-Bias Ratings across Included Studies. Note: Risk of bias was assessed using the Joanna Briggs Institute (JBI) Critical Appraisal Checklists across six domains: selection bias, performance bias, detection bias, attrition bias, confounding bias, and reporting bias. Ratings were classified as low (green), moderate (yellow), or high (red) risk. (**A**) summarizes ratings for studies examining pilots’ flight performance, and (**B**) for studies investigating cognitive factors relevant to flight performance.

Among the 31 studies investigating the association between pilots’ cognitive modules and flight performance, 27 (87.1%) were rated as high quality and 4 (12.9%) as moderate, with no studies categorized as low quality (Table 2). The summarized risk-of-bias profiles are presented in Figure 2A. The Critiplot summarizing the risk-of-bias ratings for each study across all bias domains is provided in the Supplementary Materials (Figure S10). For the 74 studies exploring the effect of aviation risk factors on pilots’ cognitive modules, 63 (85.1%) were of high quality and 11 (14.9%) were moderate (Table 2). The summarized risk-of-bias profiles are presented in Figure 2B. The Critiplot summarizing the risk-of-bias ratings for each study across all bias domains is provided in Figure S11.

Overall, most of the included studies were rated as high quality and generally exhibited low levels of selection, performance, detection, confounding, and reporting bias. However, attrition bias demonstrated relatively higher proportions of “high-risk” ratings (42% for flight performance studies and 64% for risk factor studies). This pattern likely reflects participant dropout and incomplete outcome reporting commonly observed in long-duration simulation or physiological exposure tasks, rather than deliberate methodological shortcomings.

### 3.5. Sensitivity Analyses

Across each of the individual studies being removed, the average weighted effect size remained stable (Fisher’s *z* = 0.351, ranging from *z* = 0.343 to *z* = 0.364), indicating that the overall findings of this meta-analysis are robust and not disproportionately influenced by any single study. The largest impact on the pooled effect size was observed when removing the study with the smallest original effect size (*r* = 0.134). After its exclusion, the overall effect size changed only minimally, from *r* = 0.337 to *r* = 0.348. Taken together, these results suggest that the meta-analytic conclusions are not contingent on any individual study and demonstrate satisfactory robustness.

As shown in Table S8, adding flight-measure type to the main model did not significantly improve model fit (likelihood-ratio test: χ^2^(2) = 1.77, *p* = 0.414). Information criteria further favored the more parsimonious cognitive-module-only model: AIC = −22.56, AICc = −19.04, and BIC = 4.57 for the main model, compared with AIC = −20.32, AICc = −15.34, and BIC = 11.73 for the additive model. The flight-control and situation-awareness coefficients were not statistically significant in the additive model. Therefore, adjusting for flight-measure type provided no evidence of improved model fit.

The interaction model also did not significantly improve fit relative to the additive model, χ^2^(11) = 17.13, *p* = 0.104, or relative to the main model, χ^2^(13) = 18.90, *p* = 0.126. Information criteria further favored the simpler models, particularly BIC. Several interaction terms were non-estimable because some module-by-flight-type combinations were empty or redundant. Although the interaction between working memory and situation-awareness outcomes had a *p* value close to 0.05, *b* = 0.352, SE = 0.180, *p* = 0.0503, 95% CI [−0.001, 0.705], the omnibus comparison did not support the interaction model. This coefficient was therefore interpreted as exploratory rather than as evidence of a robust moderation effect. The regression coefficients (*b*), standard errors (SE), and 95% confidence intervals for each predictor are summarized in Table S9.

Taken together, these analyses provided no evidence that including flight-measure type, either as an additive moderator or interactions with cognitive module, improved model fit. The cognitive-module-only model (main model) was therefore retained as the most parsimonious model.

### 3.6. Publication Bias Assessment Results

**Figure 3:**
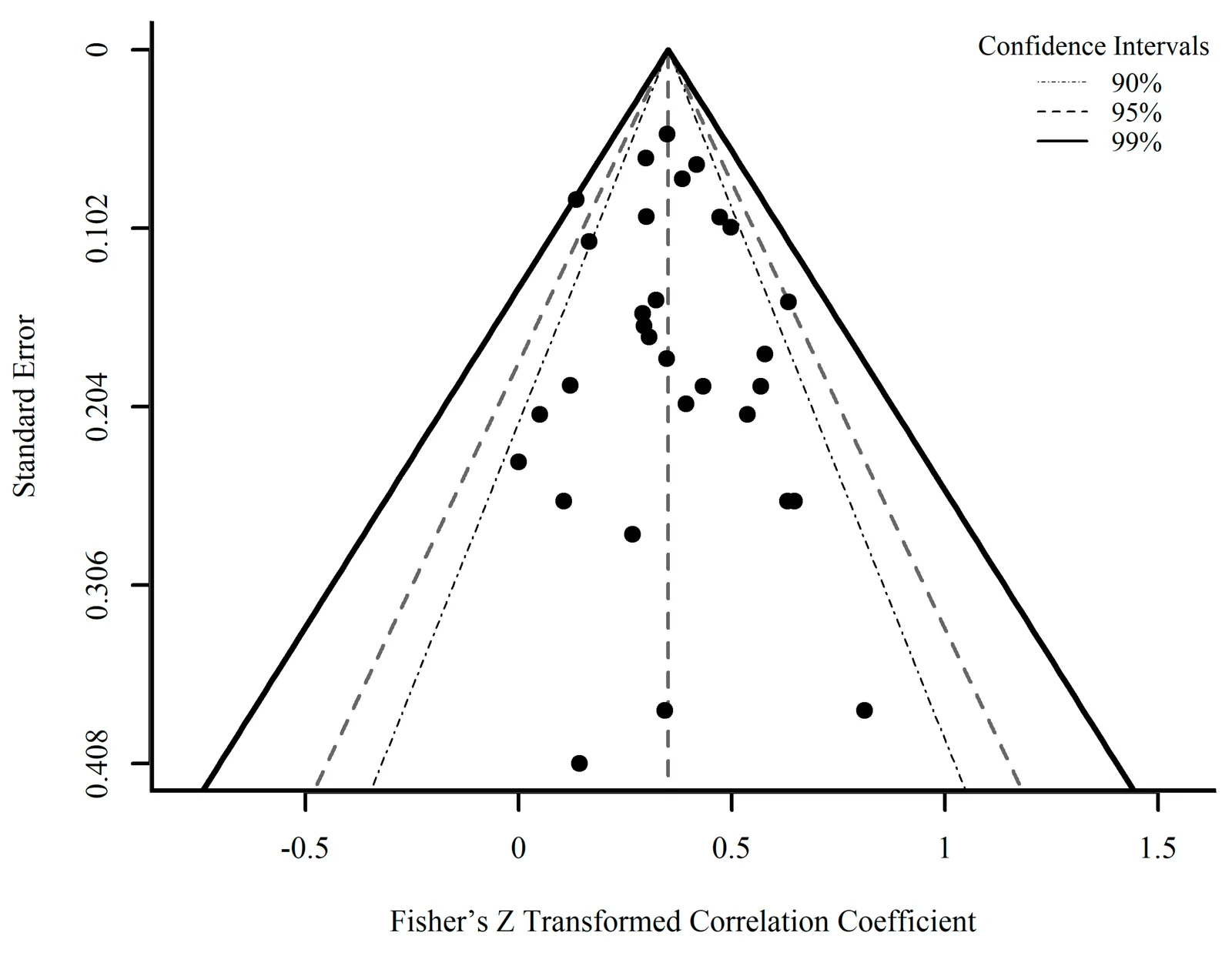
Funnel Plot of Effect Sizes (Fisher’s *z*) for the Association Between Cognitive Modules and Flight Performance. Note. Each point represents an individual effect size (Fisher’s *z*) from a single study. The vertical dashed line indicates the pooled effect size estimated from the random-effects model (*z* = 0.351). The diagonal dotted lines represent the 95% confidence interval around the pooled effect, illustrating the expected distribution of effect sizes in the absence of publication bias. The funnel plot is symmetric around the pooled effect, with most studies falling within the triangular region, suggesting no substantial small-study effects.

To assess the potential influence of publication bias, we employed multiple complementary approaches. Visual inspection of the funnel plot (Figure 3) revealed no obvious asymmetry. However, Egger’s regression test for funnel plot asymmetry was not statistically significant (*z* = 0.37, *p* = 0.713), providing no evidence of small-study effects. The trim-and-fill procedure estimated zero missing studies on the left side of the funnel plot (*k* = 0), and the adjusted pooled effect size remained essentially identical to the original estimate (original *z* = 0.351, adjusted *z* = 0.351), further supporting the robustness of the findings against publication bias. The precision effect test (PET) yielded a limit estimate of *b* = 0.334 (95% CI [0.227, 0.441]) as standard error approached zero, with a non-significant result for funnel plot asymmetry (*p* = 0.713), indicating that the observed effect was not driven by small-sample bias. Finally, Rosenthal’s fail-safe *n* was 2194, substantially exceeding Rosenthal’s criterion of 5*k* + 10 = 160 (where *k* = 30 is the number of studies), suggesting that over 2000 null studies would be needed to overturn the present findings.

Taken together, within the meta-analytic subsample of 31 studies, these supplementary checks did not reveal clear statistical evidence of funnel plot asymmetry or small-study effects. However, these findings should be interpreted with caution. With only 31 independent studies, such precision-based tests have limited statistical power to detect genuine bias. Moreover, the 31 study-level effect-size estimates remained substantially heterogeneous (null model: *Q*(86) = 209.97, *p* < 0.001, total τ^2^ = 0.0204; Table S8), which induces overdispersion in the funnel plot and violates the core assumption underlying these regression-based methods: that all dispersion is attributable solely to sampling error. These analyses, therefore, are treated as descriptive sensitivity checks for the currently included studies. In addition, the results of these analyses cannot be generalized to 93 studies including 74 reviewed qualitatively in Section 4.2.

## 4. Discussion

### 4.1. Cognitive Modules and Piloting

Beyond elevated cognitive demands, piloting is characterized by substantial variability. Cognitive demands fluctuate markedly across flight phases: cruise may allow relative relaxation, whereas approach and landing are dominated by checklists, manual control, and time-critical decision-making. Flight performance, thus, constitutes a complex ensemble of tasks. Empirical studies distill this complexity into a limited set of performance metrics to capture specific dimensions of performance, such as decision accuracy, path deviation, and speed maintenance (O’Hagan et al., 2020; Kennedy et al., 2010). By contrast, laboratory-based cognitive assessments are fundamentally designed to isolate and measure discrete cognitive modules. This methodological divergence has constrained the direct translation of cognitive findings to operational aviation contexts. The current review seeks to identify cognitive modules that exhibit robust applicability and generalizability across diverse flight-performance metrics.

**Figure 4:**
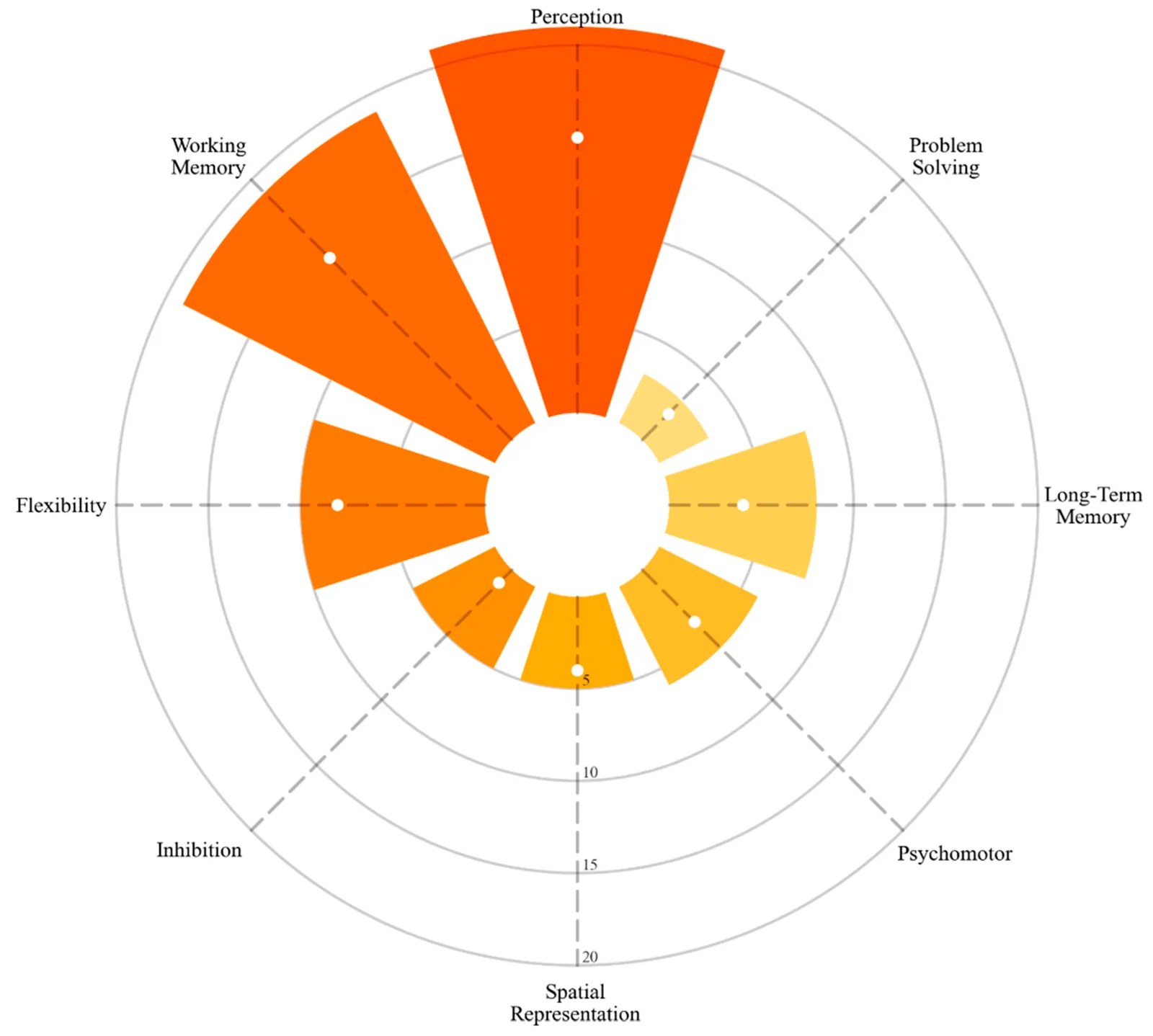
The Distribution of Eight Cognitive Modules Among Flight Performance Studies. Note. Sector height denotes the number of studies that assessed a given cognitive module in relation to pilots’ flight performance. White dots along the dashed line indicate the proportion of those studies reporting a significant association between the module and performance. The perception module was the most commonly examined (21 studies), whereas the problem-solving module was the least represented (3 studies). Because some studies evaluated multiple modules, they contribute to each relevant category, such that the summed sector heights across the eight modules exceed the total number of articles in the dataset (*n* = 31).

Across 31 studies (Table S7 in the Supplementary Document), pilots’ flight performance and cognitive modules were evaluated either separately or concurrently. The distribution of cognitive modules measured in relation to pilots’ flight performance is illustrated in Figure 4. Drawing on both empirical findings and established theoretical links, Section 3.1 identifies candidate cognitive modules based on their empirical associations with flight performance. The proposed core cognition set was then synthesized and presented in Section 4.1.

#### 4.1.1. Perception

Perception involves the awareness, organization, and ultimate identification of sensory information (Loh et al., 2004). Effective piloting and decision-making depend on continuous perceptual monitoring of multiple information channels (e.g., airspeed displays and weather radar). This perceptual demand extends across all phases of flight. Even when operations are temporarily delegated to the autopilot, pilots must sustain vigilance to detect critical instrument changes and initiate timely responses (FAA, 2021).

The meta-analysis revealed a moderate and stable correlation between the perception module and flight performance (Fisher’s *z* = 0.434, *r* = 0.41, 95% CI [0.35, 0.52], *p* < 0.001). This empirical regularity is theoretically interpretable within Endsley’s (1995) three-level model of situation awareness (SA), in which perception constitutes Level 1 situation awareness and serves as the foundational stage for detecting and encoding critical environmental cues such as altitude, airspeed, and terrain features. Without reliable perceptual input, subsequent higher-order cognitive operations, including working memory, problem-solving, and decision-making, would necessarily operate on an incomplete or inaccurate information base, thereby compromising overall flight performance. Thus, this dependency positions perception as a gateway function in the aviation cognitive chain, such that the efficiency of perceptual processing constrains the quality of all downstream cognitive operations, irrespective of variations in task complexity or automation level.

The satisfactory precision of the meta-analytic estimate further strengthens this interpretation, as it suggests that the observed perceptual demand is not an artifact of specific experimental conditions nor restricted to unusual emergency scenarios, but remains consistently detectable across routine monitoring tasks and autopilot supervision. In addition, the inclusion of perception-based tasks in regulatory fatigue management frameworks further attests to the practical recognition of this cognitive module within the regulatory system (FAA, 2013; ICAO, 2020; IATA et al., 2015). Taken together, the convergence of precisely estimated meta-analytic findings, well-established cognitive theory, and applied regulatory practice provides compelling support for including perception as a core module within the cognition set.

#### 4.1.2. Working Memory

Working memory is commonly characterized as a workspace in which information is actively maintained, updated, and manipulated to support complex mental activities (Baddeley, 1992). Within Endsley’s three-level model of situation awareness (SA), working memory does not map neatly onto any single level; rather, it functions as a transversal cognitive resource that underpins progression across all three SA levels. At Level 1, it sustains perceived information against decay and interference; at Level 2, it integrates disparate perceptual cues with knowledge retrieved from long-term memory to form a coherent understanding of the current operational state; and at Level 3 (Sohn & Doane, 2004), it enables the mental simulation and forward projection of future system states, which is essential for anticipatory decision making. In the aircraft cockpit, these processes are continuously engaged as pilots integrate fragmented sensory inputs to construct and update a coherent representation of the aircraft and its surrounding environment.

This trans-level role makes working memory a critical bottleneck in the SA cycle. When working memory is overloaded or impaired, lapses can occur at any SA level: critical cues may be lost before they can be comprehended, current situation models may fail to integrate new information, or future state projections may become inaccurate (Gutzwiller & Clegg, 2013; Stanton et al., 2009). While aviation accidents typically arise from cascading errors rather than a single cognitive failure, incidents such as the American Airlines Flight 965 crash illustrate how time pressure and information management failures can disrupt working-memory-dependent updating of critical positional data, such as terrain proximity, ultimately contributing to the accident (Idowu et al., 2022).

Consistent with its theoretical centrality, working memory was one of the most frequently examined cognitive modules in the present dataset. Our meta-analysis yielded a moderate and highly significant positive association between working memory ability and flight performance (Fisher’s *z* = 0.424, *r* = 0.40, 95% CI [0.33, 0.52], *p* < 0.001; Table 1). This association was broadly consistent across studies employing different working memory paradigms, including complex span tasks (e.g., Xie et al., 2024), *n*-back tasks (e.g., Causse et al., 2011a), and mathematical calculation (e.g., O’Hagan et al., 2020).

These findings quantitatively confirm that working memory serves as a core cognitive determinant of flight performance, not by virtue of occupying a single SA level, but by supporting the dynamic integration of perception, comprehension, and projection that defines skilled situation awareness. Taken together, the convergence of meta-analytic evidence, well-established cognitive theory, and operational relevance provides compelling support for including working memory as a core module within the proposed cognition set.

#### 4.1.3. Cognitive Flexibility

Flight operations constitute a complex ensemble of multiple concurrent tasks, requiring pilots to monitor instruments, adjust flight controls, and communicate with air traffic control simultaneously, particularly during high-workload phases such as approach and descent (Barron & Rose, 2017; Chou et al., 1996). Emergencies further impose additional demands, such as diagnosing system failures while maintaining aircraft control. The cognitive module centrally involved in these contexts is cognitive flexibility, which refers to the ability to shift between multiple mental sets, dynamically reallocate attentional resources, and update action plans in response to changing task demands (Scott, 1962; Miyake et al., 2000). Whereas perception provides the input gateway for situation awareness and working memory serves as its integration backbone, cognitive flexibility fulfills a distinct and complementary role by enabling the rapid reconfiguration of attentional focus and task priorities. This function is particularly critical in the multitask-rich environment of the cockpit, where pilots must continuously alternate between monitoring, communication, and control activities.

Operationalizations of cognitive flexibility in the reviewed literature fall into two broad categories. The first, rule-switching paradigms, typically require participants to alternate between different rules, as in the Wisconsin Card Sorting Task (Causse et al., 2011a; Kennedy et al., 2013). The second, multitasking paradigms, assess the efficiency of managing concurrent task demands, often quantified as multitasking costs under dual-task or multiple-task conditions (Guan, 2003; Griffin, 1998; O’Hagan et al., 2020; Van Benthem & Herdman, 2021a). These two approaches tap into qualitatively different aspects of flexibility and, as shown below, exhibit differential predictive validity for flight performance.

The meta-analysis revealed a clear dissociation between these two operational definitions. Multitasking measures showed a moderate and highly significant positive association with flight performance (Fisher’s z = 0.440, r = 0.41, 95% CI [0.33, 0.56], *p* < 0.001; Table 1), with a relatively small standard error indicating precise estimation. In contrast, rule-switching measures yielded a substantially smaller effect (Fisher’s z = 0.200, r = 0.20, 95% CI [0.09, 0.31], *p* = 0.0003; Table 1), and the confidence intervals of the two estimates did not overlap, suggesting that the multitasking dimension of flexibility is more strongly and consistently associated with flight performance.

This dissociation is interpretable in light of cockpit design and operational procedures. Modern aviation emphasizes standardized operating procedures and consistent display conventions, which minimize the need for rapid rule-switching during routine flight operations (FAA, 2014, 2021). In contrast, the concurrent management of multiple information streams and task goals is inherent to almost all phases of flight, from takeoff to landing, making multitasking demands a persistent feature of the operational environment. Therefore, while rule-switching ability may become critical during rare abnormal or emergency situations that require reconfiguration of goals and strategies, its contribution to overall flight performance appears limited under routine conditions.

Taken together, the convergence of meta-analytic evidence and operational logic provides compelling support for affirming the multitasking dimension of cognitive flexibility, rather than rule-switching, as a core module within the proposed cognition set for aviation performance.

#### 4.1.4. Inhibition

Inhibition refers to the suppression of dominant or prepotent responses when they conflict with current task goals (Harnishfeger, 1995). Alongside working memory and cognitive flexibility, inhibition is traditionally considered a core component of executive functions (Baggetta & Alexander, 2016; Miyake et al., 2000). However, its functional role in aviation operations remains less clear than that of the other executive components. While working memory and multitasking flexibility show robust positive associations with flight performance, inhibition may not confer the same degree of advantage, as effective piloting often requires maintaining concurrent awareness of multiple information streams rather than actively suppressing specific inputs (Causse et al., 2011a; Wickens & Alexander, 2009).

Our meta-analysis provides quantitative evidence consistent with this view. Inhibition measures yielded a small and marginally significant positive association with flight performance (Fisher’s z = 0.162, r = 0.16, 95% CI [0.003, 0.322], *p* = 0.046; Table 1). The lower bound of the confidence interval approached zero, indicating that the true effect may be negligible in some populations or contexts. Moreover, this estimate is substantially lower than those observed for perception (r = 0.41), working memory (r = 0.40), and multitasking flexibility (r = 0.41), suggesting that inhibition accounts for considerably less variance in flight performance than the other candidate modules.

The present findings suggest that, in the aviation context, the unique variance of working memory and multitasking flexibility is functionally relevant to flight performance, whereas the unique variance of inhibition appears less critical. This differential pattern is consistent with Miyake et al. (2000) unity-and-diversity framework of executive functions, which posits that while executive components share a common underlying mechanism, each also contributes unique variance to complex performance. The limited predictive utility of inhibition is theoretically interpretable in terms of cockpit engineering and operational demands. Modern flight decks are designed to aggregate and prioritize information through standardized displays, color coding, and alerting systems, thereby reducing the pilot’s need for endogenous suppression of irrelevant cues (Wickens & Alexander, 2009; FAA, 2014). Under such conditions, additional “subjective” filtering of inputs may be maladaptive, as it increases the risk of missing critical but less salient information. This interpretation is consistent with the broader cognitive science literature, which suggests that the adaptive value of inhibitory control is highly context-dependent and may be attenuated in environments where task-relevant information has already been externally structured (Miyake et al., 2000; Murphy & Creux, 2021).

It should be acknowledged, however, that certain abnormal situations may benefit from selective inhibition. For instance, when instrument malfunctions produce persistently misleading alerts, pilots may need to deliberately disregard faulty indicators and rely on cross-validation from alternative sources (e.g., computing fuel remaining from burn rates when a fuel sensor fails). In such cases, inhibitory control can serve a useful function by preventing erroneous information from corrupting situation awareness. Nevertheless, these scenarios represent exceptions rather than routine operational demands, and the overall meta-analytic evidence does not support inhibition as a general-purpose predictor of flight performance.

Taken together, the small effect size, the marginal statistical precision, and the limited theoretical alignment with routine cockpit operations indicate that inhibition does not meet the criteria for inclusion as a core cognitive module in the proposed set. It is therefore excluded from the core cognition set, while its potential role in specific emergency contexts may warrant further investigation in future research.

#### 4.1.5. Spatial Representation

Spatial representation, often operationalized through mental rotation tasks, refers to the ability to integrate fragmented spatial cues into coherent representations of objects and their orientations, thereby supporting navigation and spatial reasoning (Vandenberg & Kuse, 1978). In aviation, this ability has been hypothesized to contribute to pilots’ interpretation of aircraft attitude and spatial orientation, particularly when external visual references are limited.

The meta-analysis revealed a moderate positive association between spatial representation measures and flight performance (Fisher’s *z* = 0.344, *r* = 0.33, 95% CI [0.204, 0.483], *p* < 0.001; Table 1), with a standard error comparable to those of other modules. This effect is statistically robust and comparable in magnitude to that of included cognitive modules. Nevertheless, for the purposes of framework construction, the theoretical status of spatial representation warrants careful consideration.

Spatial representation does not map cleanly onto any single cognitive operation. Rather, it appears to emerge from the joint operation of two processes already represented in the proposed framework: perception, which encodes the spatial features of the environment, and working memory, which maintains and manipulates these spatial features into a coherent mental model. In other words, the construct of spatial representation may be characterized less as a distinct cognitive module and more as a behavioral outcome of perception and working memory acting in concert. This interpretation is consistent with evidence that mental rotation tasks draw heavily on both perceptual encoding and working memory updating (Hyun & Luck, 2007), and that their predictive power for complex performance is substantially attenuated when these constituent processes are statistically controlled.

This conceptual overlap raises a practical consideration for framework construction. The proposed framework is organized around functionally distinct cognitive operations: information intake (perception), information integration (working memory), and attentional reconfiguration (cognitive flexibility). Introducing spatial representation as a separate module would introduce redundancy, as its functional contribution can be largely accounted for by the combined roles of perception and working memory. While spatial representation may be a useful construct in specific research contexts, its inclusion as a core module would reduce the parsimony of the framework without adding a qualitatively distinct operational capability.

Taken together, despite a moderate correlation with flight performance, spatial representation is not included as an independent module in the proposed core cognitive framework. Its observed predictive utility is interpreted as reflecting the combined influence of perception and working memory, rather than a unique cognitive mechanism requiring separate representation. Future studies employing structural equation modeling or mediation analyses may further clarify the extent to which spatial representation contributes unique variance beyond these two established modules.

#### 4.1.6. Psychomotor

The psychomotor module refers to the translation of cognitive intentions into coordinated motor actions, encompassing fine motor control, hand-eye coordination, and the dynamic modulation of control inputs in response to environmental feedback (Lezak et al., 2012). In aviation, this module is engaged whenever pilots adjust control inputs to align with instrument indications or target parameters, such as maintaining glideslope during approach or tracking the runway centerline during landing.

Within the proposed framework, the psychomotor module occupies a distinct and complementary role relative to perception and working memory. Whereas perception provides the input gateway for environmental information and working memory serves as the integration backbone for decision making, psychomotor function constitutes the execution interface through which cognitive decisions are translated into precise manual actions. This sequential relationship (perception → cognition → action) positions psychomotor ability as the final link in the chain of effective piloting, rather than a redundant overlay on other cognitive modules.

Our meta-analysis provided robust empirical support for this theoretical positioning. Psychomotor measures showed a moderate and highly significant positive association with flight performance (Fisher’s *z* = 0.356, *r* = 0.34, 95% CI [0.240, 0.473], *p* < 0.001; Table 1), with a standard error comparable to that of other core modules. Importantly, the independent contribution of psychomotor beyond other cognitive modules has been demonstrated in studies that statistically controlled for perceptual and executive measures. For instance, Taylor et al. (2000) found that psychomotor performance accounted for unique variance in flight training outcomes after accounting for other cognitive predictors, suggesting that the observed association is not merely a byproduct of shared variance with perception or working memory.

The operational relevance of psychomotor function remains pronounced despite advances in cockpit automation. While automation has progressively shifted the pilots’ role from direct manual operator to supervisory manager, manual control remains indispensable during takeoff and landing, as well as in response to automation failures or unexpected flight conditions (Casner et al., 2014; FAA, 2021). The importance of psychomotor ability is particularly evident in rotary-wing aircraft, where pilots must sustain continuous manual control inputs throughout all phases of flight due to the inherent instability of helicopters (Osman et al., 2024). One investigation with helicopter pilots has developed flight-embedded psychomotor assessments for real-time cognitive monitoring, further attesting to the practical relevance of this module (McMahon & Newman, 2018).

Taken together, the convergence of theoretical distinctiveness, meta-analytic evidence, and operational necessity provides compelling support for affirming the psychomotor module as a core component within the proposed cognitive framework for aviation performance. Its inclusion ensures that the framework captures not only how pilots perceive and process information, but also how they effectively translate cognitive decisions into precise, time-sensitive actions in the cockpit.

#### 4.1.7. Long-Term Memory

Long-term memory involves the encoding, storage, and retrieval of information across extended periods (Humphreys et al., 1989). Within Endsley’s three-level model of situation awareness, it contributes mainly to Level 2 comprehension, where perceptual information is interpreted through existing knowledge, and to Level 3 projection, where previous experience guides the prediction of future system states.

A critical theoretical distinction must be made between two qualitatively different functions that fall under the scope of long-term memory. The first is static knowledge, which comprises factual and procedural information specific to a domain. In aviation, this knowledge includes meteorological principles, understanding of aircraft systems, and standard operating procedures acquired through training and experience. Such knowledge is routinely assessed through recurrent proficiency checks conducted by regulatory bodies (FAA, 2021) and lies largely beyond the focus of a cognitive architecture centered on core information-processing mechanisms.

The second and more cognitively dynamic component of long-term memory is prospective memory. This refers to the ability to remember and execute an intended action at the correct future moment or in response to a particular event, often described as “remembering to remember” (Graf & Uttl, 2001). Prospective memory differs from retrospective memory because there is typically no external cue to trigger recall. Instead, retrieval must be initiated internally in response to temporal or environmental conditions. This capacity is particularly critical in aviation, where pilots must remember to perform planned actions such as making mandatory radio calls at specific altitudes, reconfiguring systems at designated waypoints, or monitoring emerging weather conditions while attending to concurrent flight tasks (Dismukes, 2008). Empirical and incident analyses have identified failures of prospective memory as contributing factors in aviation errors, especially when workload and attentional demands are high (Dismukes, 2008).

Our meta-analysis identified a significant positive association between long-term memory measures and flight performance (Fisher’s *z* = 0.255, *r* = 0.25, 95% CI [0.137, 0.372], *p* < 0.001; Table 1), with a standard error (SE = 0.060) comparable to other cognitive modules. However, most studies included in this analysis operationalized long-term memory using retrospective recall of learned material rather than measures of prospective memory. The ecological validity of such retrospective tests for predicting real-time flight performance is therefore limited, as they fail to capture the self-initiated retrieval processes central to prospective memory in operational contexts.

Some experimental studies have demonstrated a link between prospective memory and flight performance. For instance, Van Benthem and Herdman (2020, 2021b) required pilots to remember to complete preplanned subtasks during flight, such as performing specific radio communications at predefined altitudes. Despite these findings, the available evidence focusing specifically on prospective memory, as distinct from general retrospective memory, remains sparse. Only two studies in our meta-analytic sample provided quantifiable associations between prospective memory performance and flight outcomes.

In summary, retrospective long-term memory as a general construct shows only a modest correlation with flight performance (*r* = 0.25). Nevertheless, both theoretical analysis and limited empirical findings suggest that its subcomponent, prospective memory, may play a particularly significant role in supporting flight operations. The unique cognitive demand of prospective memory, which involves self-initiated retrieval of intentions without external prompting, corresponds directly to the multitasking and attentional regulation required in piloting (Dismukes, 2008). However, given the scarcity of direct empirical evidence linking prospective memory to flight performance, current data are insufficient to recommend its inclusion as a core module within the proposed cognitive framework. Future research should focus specifically on prospective memory in operational or high-fidelity simulated flight environments.

#### 4.1.8. Problem-Solving

Problem-solving refers to the goal-directed processes of analyzing and organizing information to generate plans and guide action (Rips, 1983). In cognitive research, this module is typically operationalized through structured reasoning tasks such as the Tower of London or the One Touch Stockings Test (Robbins et al., 1994; Unterrainer et al., 2004). In aviation, similar cognitive demands arise during non-routine situations where pilots must evaluate options and select a course of action. Conceptually, the same cognitive faculty of problem-solving is engaged in both contexts.

The meta-analysis yielded a moderate positive association between problem-solving measures and flight performance (Fisher’s *z* = 0.354, *r* = 0.34, 95% CI [0.085, 0.622], *p* = 0.010; Table 1). However, this estimate is based on only three effect sizes (k = 3), and the standard error (SE = 0.137) is more than twice that of other modules, resulting in an extremely wide confidence interval with a lower bound approaching zero. This imprecision precludes a stable or reliable estimate of the problem-solving-performance relationship.

A further concern relates to construct validity. Even if a more substantial evidence base were available, the extent to which laboratory problem-solving tasks capture the processes that support operational decision-making in flight would remain unclear. Aviation problem-solving unfolds in dynamic, time-pressured environments, often involving incomplete information, team coordination, and specialized domain knowledge (Burian et al., 2005). Critically, the problem-solving construct relevant to piloting concerns the pilot’s ability to organize available tools and information into effective solutions. Standard laboratory paradigms do not approximate this capability, and the existing evidence does not permit an assessment of the gap between what these tasks measure and what pilots actually do when solving operational problems.

Taken together, the combination of a limited evidence base, imprecise estimation, and unresolved construct validity leads us to exclude problem-solving from the proposed core cognitive framework at this stage. Its conceptual relevance to aviation is not in question; rather, it is the absence of sufficient evidence for a reliable and valid measurement basis that motivates this decision.

#### 4.1.9. Summary: A Four-Module Cognition Set for Piloting

The meta-analytic evidence and theoretical analysis support the inclusion of four core cognitive modules in the proposed framework for aviation performance: Perception, Working Memory, Multitasking Flexibility (the multitasking dimension of cognitive flexibility), and Psychomotor. The selection of these modules was guided by three primary criteria:

(1) Magnitude of Effect Size: Each of the four included modules demonstrated a moderate and statistically significant correlation with flight performance, with effect sizes (Fisher’s *z*) ranging from 0.356 to 0.440 and correlation coefficients (*r*) between 0.34 and 0.41. This contrasts with modules like inhibition (*r* = 0.16), long-term memory (*r* = 0.25), and rule-switching flexibility (*r* = 0.20), which showed substantially smaller effects.
(2) Stability and Precision of the Estimate: The meta-analytic estimates for the included modules were characterized by relatively narrow confidence intervals and small standard errors, indicating high precision and consistency across studies. This robustness suggests the observed relationships are not artifacts of specific experimental conditions but represent stable, generalizable phenomena. In contrast, modules like problem-solving were excluded due to imprecise estimation (a wide confidence interval with a lower bound approaching zero) stemming from a limited evidence base (k = 3).
(3) Theoretical Parsimony and Contribution: Each included module represents a qualitatively distinct and non-redundant cognitive operation that is essential for characterizing the piloting process. Modules such as spatial representation, despite a moderate effect size, were excluded because their functional contribution is largely accounted for by included modules with a bigger effect size, and their inclusion would reduce the parsimony of the framework without adding a unique operational capability. Similarly, inhibition was excluded as its contribution appears context-dependent and less critical to the core, routine demands of the cockpit.

These four included modules do not function as independent, isolated competencies. Instead, they form an integrated, sequential, and recursive cycle that reflects the continuous flow of information processing essential for effective flight.

The process begins with the perception module, which serves as the primary input gateway. Pilots continuously monitor and detect critical sensory information from multiple channels, including instrument readings (e.g., airspeed, altitude, heading), weather radar, and external environmental cues. This foundational step is the prerequisite for all subsequent cognitive operations, ensuring that higher-order processes operate on an accurate and current information base.

The perceived information is then actively sustained, updated, and integrated by working memory. Operating as a cross-level cognitive resource, working memory maintains information against decay, integrates disparate perceptual cues with existing knowledge to form a coherent understanding of the current situation (comprehension), and enables the mental simulation of future system states (projection). In essence, working memory constitutes the integrative backbone that upholds situation awareness over time.

As information is being processed, pilots must manage the inherent multitasking demands of the cockpit. Multitasking flexibility allows for the dynamic allocation of attentional resources across concurrent tasks that define flight operations, such as monitoring instruments, communicating with air traffic control, and adjusting flight controls, while preserving focus on primary flight objectives. This function enables rapid reconfiguration of task priorities in response to changing operational demands.

Finally, cognitive decisions derived from the preceding stages must be translated into action. Psychomotor function serves as the execution interface, converting cognitive intentions into coordinated motor actions. This includes the precise manipulation of flight controls, such as the yoke, rudder, and throttle, necessary to maintain glideslope, track the runway centerline, or execute a safe landing. It forms the final link in the chain from perception to action.

Together, these four modules form a recursive and continuously active cycle of information acquisition, retention and integration, attentional coordination, and motor execution. This functional sequence is not a linear one-time process but a dynamic loop that is perpetually engaged across all phases of flight, from taxi and takeoff through cruising to approach and landing, ensuring safe and effective pilot performance (Wickens et al., 2023).

### 4.2. Cognitive Modules and Aviation-Specific Risk Factors

Section 4.1 has identified four core cognitive modules that consistently predict flight performance: perception, working memory, multitasking flexibility, and psychomotor.

**Figure 5:**
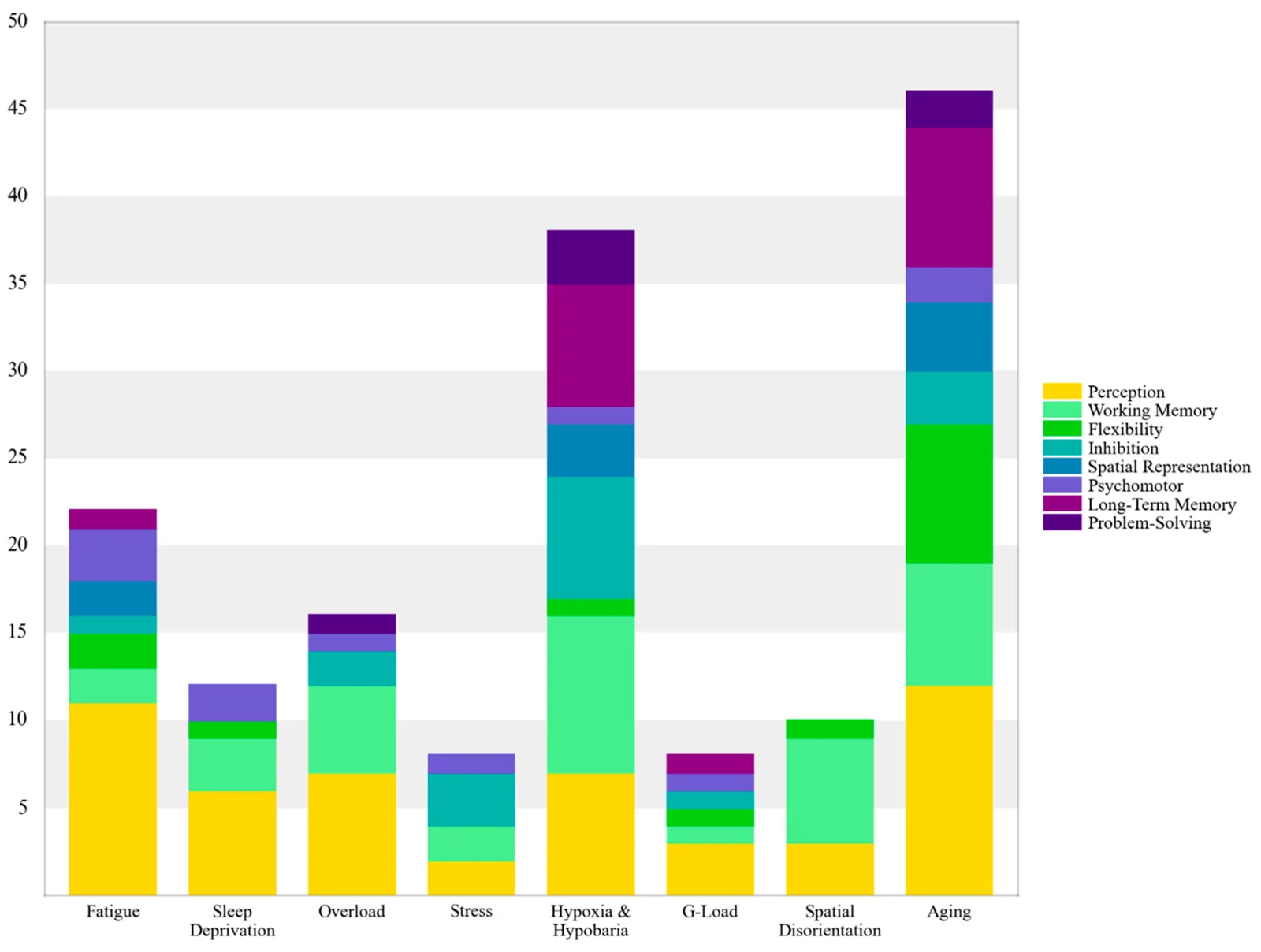
The Distribution of Eight Cognitive Modules in Aviation Risk Factors. Note. The height of each colored bar segment indicates the number of studies that assessed the corresponding cognitive module within a given topic. Because some studies evaluated multiple modules, they contribute to each relevant category, such that the summed bar segment heights across the eight modules exceed the total number of articles for a specific topic.

An additional aim of the present review was to examine how aviation-specific risk factors affect these cognitive modules. Section 4.2 summarizes 74 studies investigating the impact of occupational, environmental, and demographic factors on pilot cognition. The following sections synthesize these findings, with aggregated results presented in Table 3 and the distribution of cognitive modules across studies shown in Figure 5. Because these 74 studies were substantially heterogeneous in study design, cognitive paradigms, and outcome measures, a quantitative synthesis (i.e., meta-analysis) was not feasible. Instead, Section 4.2 presented a structured narrative synthesis following the three elements of Popay et al. (2006): (1) developing a preliminary synthesis by organizing findings by aviation-specific risk factor themes (e.g., hypoxia, fatigue, sleep deprivation); (2) exploring relationships in the data: within each theme, interpreting study findings with reference and explicitly noting conflicting evidence where present; and (3) assessing the robustness of the synthesis by considering study quality (Section 3.4). The conclusions from this section are intended to be hypothesis-generating rather than confirmatory, given the exploratory nature of the evidence base and the absence of quantitative pooling.

#### 4.2.1. Fatigue

For pilots, circadian regularity is frequently compromised by occupational demands, including jet lag, long-haul flights, sleep fragmentation, overnight duties, and irregular schedules (Caldwell, 2005). Such disruptions cumulatively engender fatigue, further causing measurable performance decrements. Through cognitive assessment, studies captured temporal dynamics of fatigue-related decline. For instance, Rosa et al. (2020) repeatedly assessed cognitive performance across an 11-h flight simulation, demonstrating progressive impairment. Portable protocols are feasible in real-world long-haul flight, for tracking cognitive status during critical phases, such as top of descent (Gander et al., 2014), layovers (Roach et al., 2012), and post-rest periods (Gander et al., 2013; Gordon et al., 2016).

Across 14 studies, fatigue produces consistent decrements in three of the four core modules. Perception is impaired through slower response speed and reduced alertness (Rosa et al., 2020; Arsintescu et al., 2022; Bartulovic et al., 2023; da Silva et al., 2021; Fletcher et al., 2022; Gander et al., 2014; Roach et al., 2012). Psychomotor function declines via degraded hand-eye coordination and fine movement (Bartulovic et al., 2023; McMahon & Newman, 2018). Cognitive flexibility suffers from increased switch costs and impaired task-set reconfiguration (Gordon et al., 2016; Rabinowitz et al., 2009). Notably, current evidence does not consistently support fatigue-related impairments in working memory, long-term memory, inhibition, or spatial representation (Gordon et al., 2016; Rosa et al., 2020). For example, Bartulovic et al. (2023) reported working memory decrements with longer duty durations, whereas Rosa et al. (2020) reported no decline. The effect of fatigue on problem-solving remains unexplored.

#### 4.2.2. Sleep Deprivation

Aviation systems emphasize fatigue management to safeguard flight safety (FAA, 2013; ICAO, 2020; IATA et al., 2015). Nonetheless, certain missions require sustained overnight operations, making sleep deprivation unavoidable. In these scenarios, effective risk management depends on identifying the onset of cognitive degradation under prolonged wakefulness.

Within the reviewed studies, 7 studies tracked pilots’ cognitive modules across periods of enforced wakefulness ranging from 17 h (Wingelaar-Jagt et al., 2023b) to 62 h (Caldwell et al., 2004). During these studies, subjects cycled between rest periods, flight tasks, and non-flight duties. Measurable cognitive decline emerged after approximately 18–21 h of continuous wakefulness (Caldwell et al., 2004; O’Hagan et al., 2020; Russo et al., 2004; Lopez et al., 2012). Notably, the decrements in working memory precede the decrements in flight performance and perception, indicating that the resource-intensive module (working memory) shows greater sensitivity to sleep loss (O’Hagan et al., 2020; Russo et al., 2004). Methodologically, these studies parallel fatigue research by employing repeated assessments to capture cognitive trajectory over time. Findings across the three affected modules roughly mirror fatigue-related deterioration, including Perception, Psychomotor, and Working Memory (Lopez et al., 2012; Caldwell et al., 2004; O’Hagan et al., 2020; Wingelaar-Jagt et al., 2023b; Russo et al., 2004; Algranati et al., 2024). Evidence remains absent for other modules, particularly cognitive flexibility, which is both sensitive to fatigue and flight performance (Bednarek et al., 2019; Yesavage et al., 2011).

#### 4.2.3. Overload

Workload varies markedly across flight phases. Takeoff and landing, in particular, demand intensive attention and execution of complex time-critical procedures (Kantowitz & Casper, 1988). Given the finitude of cognitive resources, increasing workload predictably degrades performance (Loft et al., 2023). Cognitive assessments have been adopted to elucidate how pilots allocate and manage these resources under load.

A common methodology embeds assessments as secondary tasks within flight scenarios, such as auditory target detection (Zakay & Shub, 1998), where prolonged reaction times and increased false alarms indicate resource depletion under high workload conditions. This framework consistently demonstrated that demanding flight scenarios occupy more available cognitive resources, thereby degrading secondary-task performance (Dehais et al., 2019; Ayala et al., 2024; Zakay & Shub, 1998). Beyond passively monitoring residual resource, embedded assessments could manipulate workload to quantify its impact on primary-task performance. Both manipulations reveal a significant decline in perception (Peng et al., 2022), problem-solving (Causse et al., 2017), flexibility (Hernandez-Sabate et al., 2022; Causse et al., 2024), and a reduction in peripheral awareness (Williams, 1995). Such implementations also expose individual differences in workload resilience (Caldwell, 2005), with evidence indicating that pilots with better inhibition performance managed high-demand scenarios more effectively (Dehais et al., 2019). Overall, the three modules of the cognition set (perception, working memory, and cognitive flexibility) exhibited negative responses under overload conditions. As for the psychomotor module, no evidence supports its contribution to capturing pilots’ workload in aviation (Galant-Gołębiewska et al., 2020).

#### 4.2.4. Stress

Aviation exposes pilots to a broad array of stressors beyond irregular schedules, sleep deprivation, and excessive workload. Experimental studies have elicited other perceived stress through social evaluative pressure (Causse et al., 2024), anticipatory stress (Leino et al., 1999), unexpected startle stimuli (Deniel et al., 2023), and the flight task itself (Combs et al., 2021).

Individual stress responses are reliably indexed by physiological markers, including cortisol (Combs et al., 2021; Leino et al., 1999), cerebrovascular oxygenation (Causse et al., 2024), and cardiovascular dynamics (Pattyn et al., 2014). Beyond physiological markers, stressors affect pilots’ cognitive state. Comparisons of pre- and post-stressor performance indicate that social pressure enhances the perception and flexibility modules of pilots (Causse et al., 2024), while acute stress tends to improve pilots’ working memory performance (Liang et al., 2024). Interestingly, among expert pilots, neither task complexity nor timepoint (day vs. night operations) altered cognitive inhibition, despite concomitant fluctuations in cortisol (Combs et al., 2021). For cadet pilots, cognitive state is susceptible to flight training and evaluative wording (Pattyn et al., 2014; Leino et al., 1999).

Consistent with the previous three sections, these 7 studies used the four cognitive modules in the proposed set to measure aviation factors’ effects on pilot cognition. However, stress produced both beneficial and adverse effects on cognitive modules, depending on the context, stressor type, and expertise level. Further research is needed to clarify the effect of diverse stressors in aviation contexts, with particular emphasis on emergencies and startle responses (Diarra et al., 2023; Causse et al., 2025). In addition, current evidence also points to substantial individual differences in stress reactivity (Roos et al., 2017; Morgan et al., 2013; Lin et al., 2020).

#### 4.2.5. Hypoxia & Hypobaria

In the high-altitude environment of aircraft operations, low oxygen concentration (hypoxia) and low atmospheric pressure (hypobaria) are potential risk factors that may cause both acute and chronic physiological effects, including tissue hypoxia symptoms and decompression sickness (Jersey et al., 2010; Gradwell & Rainford, 2016). To simulate these hypoxic and hypobaric conditions, studies commonly employ hypobaric chambers with a wide range of altitude settings, spanning from 2438 m (8000 ft, the maximum altitude for commercial aircraft; Thropp & Buza, 2019) to 21,336 m (70,000 ft, operational altitude of U-2 reconnaissance missions; McGuire et al., 2016). Cognitive assessments are administered under altitude profiles tailored to aircraft type and pilot population to identify hypoxia-related cognitive decline.

At the altitudes of commercial aircraft (from 2438 to 4572 m), findings are mixed. Specifically, regarding cognitive inhibition, diverse altitude-induced impacts occurred, including improved (Bouak et al., 2019), intact (Legg et al., 2016), and impaired (Thropp & Buza, 2019) performance. Other cognitive modules remained intact in these settings, including long-term memory, working memory, spatial representation, and problem-solving (Bouak et al., 2018; Legg et al., 2016). At higher exposures (roughly 3810–7500 m), impairments are consistent: more memory retrieval errors (long-term memory; Nation et al., 2017; Bustamante-Sanchez et al., 2019; Bouak et al., 2018), diminished short-term processing capacity (working memory; Aebi et al., 2020; Ilbasmis, 2024; Wen et al., 2020; Vacchiano et al., 2008), greater susceptibility to interference (inhibition; Takács et al., 2017; Asmaro et al., 2013); worse logical reasoning performance (problem-solving), and elevated task-switching costs (flexibility; Asmaro et al., 2013; Peacock et al., 2017). At altitudes approaching 10,000 m, impairments in working memory have been observed (Malle et al., 2013). Moreover, studies of U-2 pilots operating at extreme hypobaria (~21,336 m) under nonhypoxic conditions documented broad impairments spanning perception, long-term memory, problem-solving, and spatial representation (McGuire et al., 2014; McGuire et al., 2016).

Overall, cognitive impairments are most consistently observed at altitudes above approximately 3810 m (12,500 ft), with pronounced effects on working memory and cognitive flexibility. By contrast, lower-level modules (perception and psychomotor) show limited sensitivity under comparable conditions (Nation et al., 2017; Bustamante-Sanchez et al., 2019; Asmaro et al., 2013; Takács et al., 2017). Investigating the specific contributions of hypoxia versus hypobaria, particularly at extreme altitudes, remains an important priority. For example, Aebi et al. (2020) isolated hypoxia-related decrements at 5486 m (18,000 ft), whereas McGuire and colleagues demonstrated hypobaric effects at 21,336 m (70,000 ft) under adequate oxygenation (McGuire et al., 2014, 2016). Individual differences in altitude tolerance, both across aircraft types and among pilots of identical aircraft, also merit attention (Bustamante-Sanchez et al., 2019; Nation et al., 2017).

#### 4.2.6. G-Load

Aircraft maneuvers impose gravitational loads (G-loads) of varying magnitude and vector on pilots, with the load directed from head to feet (+Gz) posing particular risk. The +5 Gz redistributes blood from the brain to the lower extremities, leading to cerebral hypoperfusion and potentially causing G-Induced Loss of Consciousness (G-LOC; Green, 2006).

Five studies investigated cognitive modules under +Gz exposures ranging from +3 to +7 Gz. Some documented cognitive performance during G-load exposure (e.g., Tripp et al., 2006), whereas others are documented per- and post-performance (e.g., Biernacki et al., 2013). The +Gz-induced decrements include worse reaction speed and accuracy (perception; Truszczynski et al., 2014), increased susceptibility to incongruent information (inhibition), and higher costs of multitasking (flexibility; Dalecki et al., 2010). Furthermore, as pilots approach G-LOC, calculation-based performance (working memory) degrades before tracking performance (psychomotor; Tripp et al., 2006). Within one minute of recovering from G-LOC, both calculation and tracking efficiency remained significantly impaired (Tripp et al., 2006). Even without lapsing into G-LOC, memory deficits persisted after G exposure (Biernacki et al., 2013). In contrast, some observed post-exposure enhancements in the perception module, particularly among younger (<34 years) and taller pilots, potentially reflecting transient arousal (Biernacki et al., 2013; Ercan & Gunduz, 2020).

Despite the limited number of studies, the proposed four cognitive modules (perception, working memory, psychomotor, and flexibility) were able to detect +Gz-induced deteriorations in pilots. Furthermore, they can also be applied to capability evaluations related to G-load effects (e.g., acceleration tolerance in Truszczynski et al., 2014).

#### 4.2.7. Spatial Disorientation

Spatial disorientation (SD) refers to the failure to accurately perceive an aircraft’s attitude, position, and motion relative to the gravitational vertical (Benson & Stott, 2006). SD commonly arises when pilots prioritize unreliable external visual cues without adequately cross-checking primary flight instruments (Balaj et al., 2019). Such misperceptions degrade flight control and instrument interpretation, potentially leading to aviation incidents (Gibb et al., 2011).

Within the present review, seven studies investigated SD through a cognitive lens. For inducing SD in simulation, investigators adopted disoriented flight scenarios that elicited canonical illusions with either vestibular or visual origins. Experimental paradigms often constrained access to key attitude information (e.g., removing the attitude direction indicator), and required pilots to complete flight tasks under these misleading conditions (Balaj et al., 2019).

Performance on cognitive assessments could predict pilots’ susceptibility to disorientation illusions. Visual illusions had a lesser impact on the pilots equipped with superior working memory and flexibility (Bednarek et al., 2019). Cognitive assessments also served as embedded secondary tasks within oriented and disoriented flight scenarios, additionally requiring pilots to recognize (perception) and process (working memory) auditory stimuli during flight tasks (Lewkowicz et al., 2019). Across such paradigms, disorientation conditions are associated with cognitive performance decrements (Webb et al., 2012; Stróżak et al., 2018) and were interpreted as negative impacts of disorientation illusions, aligning with the theory of Gresty et al. (2003). However, secondary-task demands inherently consume resources and can themselves degrade flight performance under SD (Lewkowicz et al., 2018; Lewkowicz et al., 2019). In contrast, the decrements of disoriented conditions are less universal: only two of six illusion scenarios (leans and circle-to-land illusions) reliably impaired flight performance. The possibility cannot be excluded that preexisting cognitive constraints (e.g., limited capacity for cross-validation of instrument and sensory cues) may increase vulnerability to SD, rather than SD illusions directly impairing cognition.

In either theory, omission of critical information emerges as a proximal mechanism in SD-related incidents, which explains why existing studies have focused on perception, working memory, and flexibility modules that align precisely with pilots’ processing of information. Future work could examine individual differences in spatial perception efficiency and cognitive style, as suggested by Bednarek et al. (2019).

#### 4.2.8. Aging

Pilots are subject to the normative cognitive changes associated with aging (Salthouse, 2009). Across multiple studies, older pilots exhibit reliable decrements relative to younger counterparts in perception, long-term memory, working memory, and cognitive flexibility (Tsang, 1998; Hardy et al., 2007; Causse et al., 2011a; Causse et al., 2019; Taylor et al., 2000; Taylor et al., 2011; Yesavage et al., 2011; Adamson et al., 2010b; Arikan et al., 2018; Kennedy et al., 2013; Kennedy et al., 2010; Kennedy et al., 2015; Morrow et al., 2003; Audu et al., 2021; Van Benthem & Herdman, 2020, 2021a). Findings for inhibition and psychomotor modules are fewer, though most reported age-related decline (Hardy et al., 2007; Causse et al., 2011a; Arikan et al., 2018; Taylor et al., 2000). Evidence for problem-solving and spatial processing is mixed, with reports of both intact and impaired performance in older pilots (Causse et al., 2011a; Causse et al., 2019; Tsang, 1998; Morrow et al., 2003; Arikan et al., 2018; Kennedy et al., 2010).

Most studies on aging in pilots are cross-sectional, with relatively few adopting longitudinal designs. Given the lack of consensus on age thresholds in the literature, which span roughly 40–60 years (Adamson et al., 2010b; Hardy et al., 2007; Kennedy et al., 2015), it may be beneficial to consider establishing self-referenced cognitive baselines. Such baselines would enable within-person detection of aging rather than reliance on rigid cutoffs that classify all pilots beyond a threshold as “old.” Longitudinal and self-referenced monitoring would also facilitate identification of non-age factors that modulate cognitive state over time (e.g., pandemic-related stress, childbirth, family demands, nutritional status), thereby supporting more comprehensive risk management and targeted interventions.

#### 4.2.9. Summary: Patterns of Cognitive Impairment

**Figure 6:**
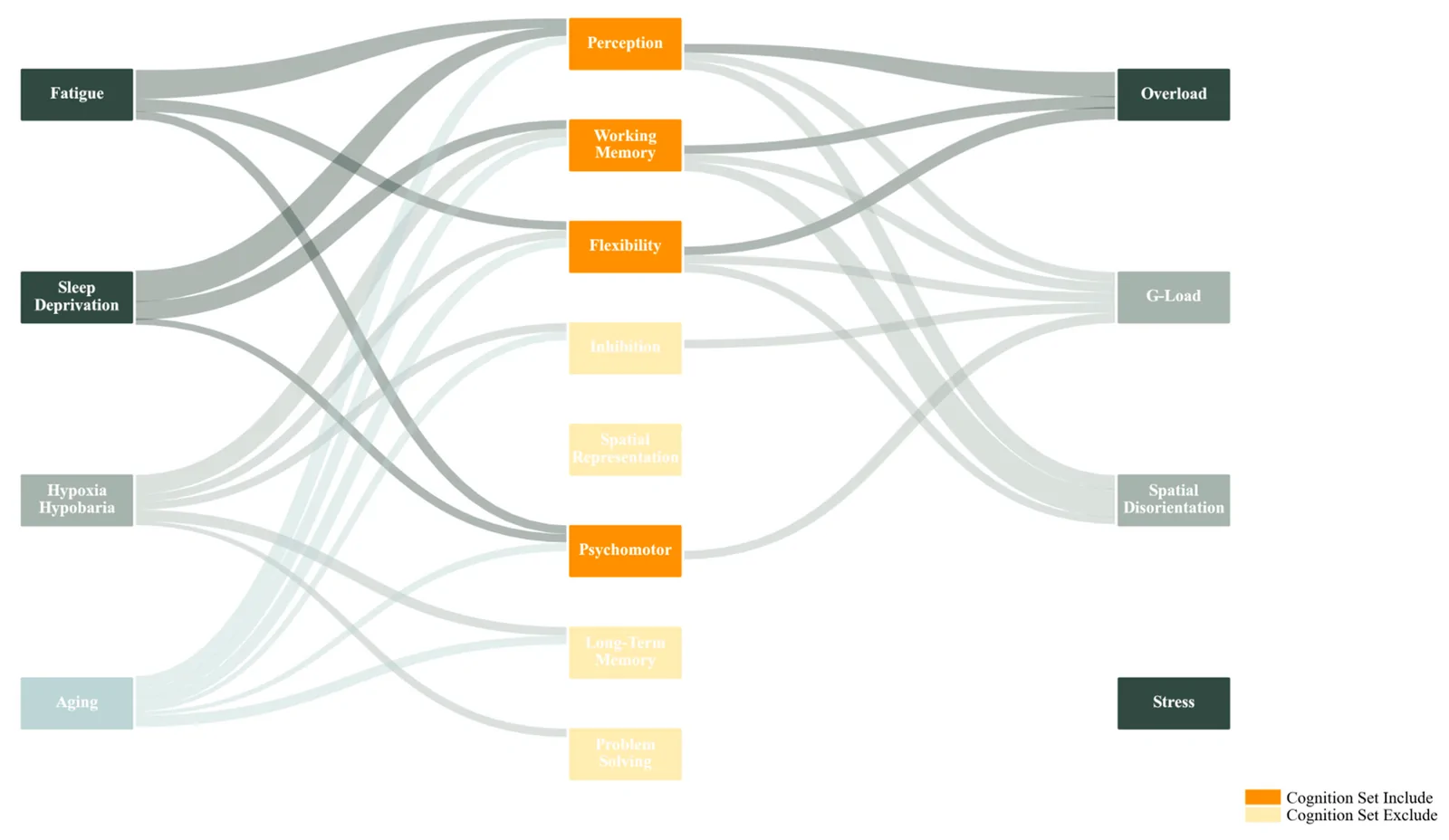
Empirical Associations Between Aviation Factors and Cognitive Modules. Note. The eight aviation factors summarized in Section 3.2 are displayed on the left and right. The central panel lists the eight cognitive modules reviewed in Section 2; four are included in the piloting-specific cognition set, and four are provisionally excluded. A connecting line between a factor and a cognitive module indicates empirical evidence that the factor’s impact is observable in the corresponding cognitive performance (and vice versa). Line width encodes the relative weight of the evidence, with wider connections representing a larger proportion of supporting studies.

Section 4.2.1, Section 4.2.2, Section 4.2.3, Section 4.2.4, Section 4.2.5, Section 4.2.6, Section 4.2.7 and Section 4.2.8 reviewed evidence linking a broad range of aviation-specific risk factors to measurable changes in cognitive performance. These stressors include occupational factors, environmental factors, and demographic factors. Stress (reviewed in Section 4.2.4) was excluded from module-level synthesis because the evidence is limited in quantity and heterogeneous in stress manipulation, precluding reliable mapping onto any cognitive module set. All outcomes have been visualized in Figure 6. Across these domains, a recurring pattern emerges: perception, working memory, psychomotor, and cognitive flexibility (bright orange blocks in Figure 6) are consistently impaired across nearly all aviation risk factors reviewed.

For perception, deficits (primarily slower response speed and reduced alertness) are documented under fatigue (Rosa et al., 2020; Arsintescu et al., 2022), sleep deprivation (Lopez et al., 2012; Caldwell et al., 2004), overload (Peng et al., 2022), +Gz load (Truszczynski et al., 2014), spatial disorientation (Webb et al., 2012; Stróżak et al., 2018), extreme hypobaria (McGuire et al., 2014, 2016), and aging (Tsang, 1998; Hardy et al., 2007; Causse et al., 2019). The only notable exception is moderate-altitude hypoxia (3810–7500 m), where perception shows limited sensitivity compared to higher-order modules (Nation et al., 2017; Bustamante-Sanchez et al., 2019).

Working memory shows similar vulnerability profile. It is consistently impaired by fatigue (though with some mixed findings; Bartulovic et al., 2023 vs. Rosa et al., 2020), sleep deprivation (O’Hagan et al., 2020; Russo et al., 2004), overload (Dehais et al., 2019), hypoxia above 3810 m (Aebi et al., 2020; Ilbasmis, 2024), +Gz load (Tripp et al., 2006), spatial disorientation (Lewkowicz et al., 2019), and aging (Kennedy et al., 2010, 2013; Van Benthem & Herdman, 2020). Under extreme hypobaria, working memory still shows evidence of impairment (McGuire et al., 2014, 2016).

Cognitive flexibility (indexed by task-switching costs and multitasking efficiency) is similarly broadly sensitive. Impairments are documented under fatigue (Gordon et al., 2016; Rabinowitz et al., 2009), overload (Hernandez-Sabate et al., 2022; Causse et al., 2024), hypoxia above 3810 m (Asmaro et al., 2013; Peacock et al., 2017), +Gz load (Dalecki et al., 2010), spatial disorientation (Bednarek et al., 2019), and aging (Kennedy et al., 2015; Van Benthem & Herdman, 2021a). Sleep deprivation studies have not systematically examined flexibility despite its known sensitivity to fatigue (Bednarek et al., 2019; Yesavage et al., 2011), representing a gap in the literature.

In contrast to the three modules above, psychomotor function shows a narrower sensitivity profile. Consistent impairments are documented under fatigue (Bartulovic et al., 2023; McMahon & Newman, 2018), sleep deprivation (Caldwell et al., 2004; Wingelaar-Jagt et al., 2023b), +Gz (Tripp et al., 2006), and aging (Hardy et al., 2007; Taylor et al., 2000). However, psychomotor shows limited or no evidence of impairment under overload (Galant-Gołębiewska et al., 2020), hypoxia at moderate altitudes (Nation et al., 2017; Bustamante-Sanchez et al., 2019), spatial disorientation, or extreme hypobaria. This narrower profile suggests that psychomotor deficits are primarily associated with risk factors that either directly affect motor pathways (e.g., +Gz), reduce global alertness and processing speed (e.g., fatigue, sleep deprivation), or compromise general neurological integrity (aging), rather than being a universal consequence of cognitive compromise.

Across diverse contexts, the modules of perception, working memory, cognitive flexibility, and psychomotor function appear to provide the most consistent indicators of pilots’ changing cognitive states under varying risk factors. A comprehensive synthesis of aviation-specific factors demonstrates that these domains are among the most frequently and robustly affected areas of performance, spanning fatigue, sleep deprivation, task overload, hypoxia, +Gz load, spatial disorientation, and aging. Importantly, this pattern does not merely reflect their vulnerability to impairment, but rather their high sensitivity to fluctuations in functional state as environmental and physiological demands vary.

The convergence of impairments observed across perception, working memory, cognitive flexibility, and psychomotor domains under multiple aviation-specific risk factors may be understood through the framework of front cortical sensitivity to physiological and neurochemical stress. These cognitive modules rely heavily on prefrontal and frontoparietal networks responsible for top-down control, attentional allocation, and sensorimotor integration. Arnsten (2009, 2015) demonstrated that such prefrontal circuits are particularly vulnerable to fluctuations in catecholaminergic signaling induced by stress, fatigue, hypoxia, and other challenges to homeostasis. Moderate levels of catecholamines (e.g., norepinephrine and dopamine) facilitate efficient prefrontal processing, but excessive or insufficient activation disrupts synaptic activity within these networks, leading to transient losses in cognitive stability and executive coordination. As these same neuromodulator pathways are affected by most operational stressors encountered in aviation—including altered oxygenation, high workload, and sleep deprivation—the cognitive domains most dependent on prefrontal regulation manifest the most consistent performance decrements. This neurobiological mechanism provides a unifying explanation for why decrements in perception, working memory, cognitive flexibility, and psychomotor coordination emerge recurrently across heterogeneous risk conditions.

While the four broadly vulnerable modules capture impairments across most risk factors, certain conditions require monitoring additional cognitive modules (faint yellow block in Figure 6).

Long-term memory is a notable example. It shows consistent impairment under hypoxia above 3810 m (Nation et al., 2017; Bustamante-Sanchez et al., 2019; Bouak et al., 2018), extreme hypobaria (McGuire et al., 2014, 2016), and aging (Causse et al., 2011a; Taylor et al., 2011; Yesavage et al., 2011). Under fatigue, sleep deprivation, overload, +Gz, and spatial disorientation, long-term memory either shows inconsistent effects or has not been systematically examined. For high-altitude operations or aging pilot populations, therefore, an assessment battery limited to the core four modules would miss significant cognitive changes in memory consolidation and retrieval.

Inhibition is another module that becomes relevant under specific risk factors. It is reliably impaired by +Gz (Dalecki et al., 2010), aging, and hypoxia above 3810 m (Takács et al., 2017; Asmaro et al., 2013), with mixed evidence under overload. For other factors, inhibition has not been systematically studied.

#### 4.2.10. Differential Sensitivity of Cognitive Modules

Beyond identifying which cognitive modules are affected by aviation risk factors, the reviewed evidence reveals systematic differences in when and how severely different modules are impaired. A hierarchical vulnerability emerges from the literature. Across multiple risk factors, resource-intense modules (working memory and cognitive flexibility) consistently show greater and earlier impairment than lower-level modules such as perception and psychomotor function. This hierarchical pattern is supported by converging evidence from several domains.

Hypoxia provides the clearest cross-sectional evidence. At altitudes above approximately 3810 m (12,500 ft), impairments are consistently observed in working memory and cognitive flexibility, whereas lower-level modules (perception and psychomotor) show limited sensitivity under comparable conditions (Nation et al., 2017; Bustamante-Sanchez et al., 2019; Asmaro et al., 2013; Takács et al., 2017). That is, the same hypoxic insult that reliably degrades working memory leaves basic perceptual speed relatively intact.

Sleep deprivation adds a temporal dimension to this hierarchy. Decrements in working memory precede decrements in both flight performance and perception (O’Hagan et al., 2020; Russo et al., 2004). Working memory is compromised first; lower-level sensory and motor processes follow later. This temporal ordering suggests that higher-order modules serve as early indicators of accumulating sleep pressure, offering a window for intervention before operational performance degrades.

A similar pattern appears under +Gz exposure, again with a temporal ordering. As pilots approach G-induced loss of consciousness (G-LOC), calculation-based working memory tasks cease before tracking-based psychomotor tasks (Tripp et al., 2006). Within extreme condition, more cognitively demanding tasks (calculation) show greater vulnerability at lower G-levels than less demanding tasks (tracking).

Taken together, these three lines of evidence converge on a consistent finding: resource-intense cognitive modules are both more sensitive to aviation risk factors and decline earlier than lower-order modules. Working memory, in particular, consistently emerges as a “canary in the coal mine”, a sensitive early indicator of cognitive compromise across multiple aviation-specific risk factors. This pattern is consistent with cognitive resource theory (Kahneman, 1973), which conceptualizes attention and cognitive processing as drawing from a limited pool of resources. Operations that require active maintenance, updating, or task-switching consume more resources than simpler perceptual detection or automated psychomotor responses. Consequently, under conditions of resource depletion (e.g., fatigue, high workload, hypoxia), resource-intensive modules show earlier and more pronounced impairment.

#### 4.2.11. Quantifying Individual Differences Through the Cognition Set

Beyond group-level patterns, the proposed cognition set also captures individual differences in vulnerability to aviation risk factors. Some maintain cognitive performance under conditions that impair others, and these differences are measurable through the same four core modules.

Hypoxia provides a clear example. Under identical altitude exposure, some pilots maintain cognitive performance while others show significant impairment. This variability occurs both across aircraft types and among pilots flying the same aircraft (Bustamante-Sanchez et al., 2019; Nation et al., 2017). This variability suggests that population-level “safe altitude” thresholds may be insufficient; some individuals require more conservative limits. +Gz exposure reveals another source of individual differences. Younger pilots (<34 years) and taller pilots showed post-exposure enhancements in perception, possibly reflecting transient arousal, whereas others did not (Biernacki et al., 2013; Ercan & Gunduz, 2020). Age and anthropometric factors, therefore, moderate the cognitive response to gravitational stress—though the mechanisms remain unclear. Spatial disorientation demonstrates that cognitive capacity itself moderates vulnerability. Pilots with superior working memory and flexibility are less susceptible to visual illusions (Bednarek et al., 2019). Here, the four-module set does not merely detect impairment; it predicts who is most at risk before exposure.

Group-level sensitivity patterns (e.g., “working memory declines under hypoxia”) are useful for system design and status monitoring, but they are insufficient for individual-level monitoring. A pilot whose performance declines from their own baseline by one standard deviation may be at risk even if their absolute performance remains within the population’s normal range. The limitations of population norms are further illustrated by aging research, where the definition of “older pilot” varies widely across studies, ranging from 40 to 60 years (Adamson et al., 2010b; Hardy et al., 2007; Kennedy et al., 2015). A rigid cutoff that classifies all pilots above a certain age as “old” suffers from the same limitation as assuming that all pilots remain cognitively intact below a specific altitude; both ignore substantial interindividual variability and provide limited actionable information for individual pilots.

### 4.3. Future Directions

As the reviewed evidence reveals systematic differences in how different cognitive modules respond to aviation risk factors, a natural and empirically grounded hypothesis emerges for future investigation: cognitive monitoring systems could achieve higher diagnostic sensitivity by prioritizing the more sensitive modules as primary indicators of pilot state degradation. For threshold calibration, for example, establishing the G-level at which G-LOC onset occurs, or the altitude above which cognitive performance becomes significantly compromised, working memory and flexibility assessments offer a compelling rationale. The superior sensitivity of these modules thus provides a promising basis for establishing safety thresholds with greater precision than lower-level measures.

The differential sensitivity patterns also open a promising avenue connecting cognitive assessment with the broader KSAO (Knowledge, Skills, Abilities, and Other characteristics) framework widely used in personnel selection and training. While KSAO models have traditionally treated cognitive abilities as static predictors of training outcomes, our findings suggest that the same abilities, particularly working memory and flexibility, exhibit dynamic vulnerability to aviation-specific risk factors (e.g., hypoxia, fatigue, +Gz). This raises an untested but consequential question: do individuals with higher baseline cognitive abilities also demonstrate greater resilience to risk-factor-induced degradation? If so, KSAO-based selection criteria might inadvertently serve as proxies for physiological tolerance, and training programs could be tailored not only to build knowledge and skills but also to inoculate against state-dependent cognitive vulnerability. Conversely, if baseline ability and stress-induced decline are orthogonal, then monitoring becomes the primary lever for managing risk. These competing hypotheses merit systematic investigation, as they carry distinct implications for pilot selection, training design, and operational risk management.

### 4.4. Limitations

Several limitations of the present review should be acknowledged to guide interpretation of the findings and future research directions.

(1) Methodological constraints related to the search strategy may have influenced the comprehensiveness of the evidence base. The review was limited to three major databases and restricted to full-text articles published in English. This restriction may have led to the omission of grey literature and non-English studies not indexed within these sources, thereby introducing potential publication bias and limiting the global representativeness of the findings.
(2) Procedural limitations arose from the timing of the meta-analytic component, which was incorporated midway through the review process. Consequently, study selection and cognitive module classification were not conducted entirely under PRISMA guidelines. Paradigm-module classification decisions were made jointly by two reviewers through consensus discussion and reference to the literature. However, because systematic documentation was not maintained during the initial classification phase, inter-rater agreement, such as Cohen’s κ, could not be formally quantified retrospectively. In addition, the present review was not preregistered on a publicly available platform.
(3) Limitations inherent to the data-driven approach underpinning the cognitive module framework must be recognized. The synthesis relied on available empirical evidence rather than testing a predefined theoretical model. As is common in semi-standardized meta-analyses, some theoretically meaningful dimensions were underrepresented in the literature. For instance, language processing was rarely examined empirically, though it is an ability that is theoretically vital to flight operations, especially for ensuring communication efficiency among pilots, crew, and air traffic control. In the absence of sufficient data, meaningful discussion of such dimensions remains constrained. As a result, the proposed cognitive set reflects the contours of the existing evidence rather than a fully comprehensive theoretical account of the cognitive architecture underlying piloting.
(4) Variability in the meta-analytic evidence base also warrants caution. The number of contributing studies differed considerably across cognitive modules, ranging from as few as three studies for problem-solving to twenty for perception. For modules with limited available studies, the pooled estimates should therefore be interpreted cautiously. Importantly, the absence or exclusion of certain modules likely reflects the current state of the literature rather than a definitive statement about their lack of relevance.
(5) Assumptions embedded in the analytic framework should be considered. The meta-analytic model treated cognitive modules as independent predictors, an assumption that may oversimplify the complex, interactive, and cascading relationships among cognitive processes that occur in actual cockpit settings. In addition, exclusions of modules such as spatial representation and problem-solving were informed by conceptual overlap and unresolved construct validity issues rather than direct statistical tests of incremental validity. Future research employing more comprehensive cognitive batteries and structural equation modeling is needed to disentangle the unique contributions of these interrelated constructs. It should also be emphasized that the exclusion of specific modules does not imply their general irrelevance to complex task performance; rather, these conclusions are specific to the aviation context and its operational demands.
(6) Although the present review aggregated multiple effect sizes within each study before conducting publication-bias tests, the small number of independent studies (*k* = 31) and the considerable heterogeneity among them (e.g., *Q*(86) = 209.97, *p* < 0.001 in the null model; Table S8) fundamentally limit the interpretability of these tests. The overdispersion induced by substantial between-study variation violates the distributional assumptions of Egger’s regression test, trim-and-fill procedure, and precision effect test (PET). Thus, their point estimates and *p*-values are unreliable as definitive evidence for or against publication bias. The present review therefore treats them as exploratory diagnostics rather than confirmatory inferences.
(7) The methodological distinction between Section 4.1 and Section 4.2 should be recognized. The conclusions presented in Section 4.1 were informed by quantitative meta-analytic estimates, as the included studies were sufficiently homogeneous to permit pooled effect-size calculations. In contrast, in Section 4.2, narrative synthesis was required because of the substantial heterogeneity in study design, measurement paradigms, and outcome measures across these 74 studies, which precluded meaningful statistical aggregation. To impose structure on this qualitative synthesis, the current review organized the findings by aviation-specific risk factor and cognitive module. Referring to the quality appraisal scores from Section 3.4, all 74 studies were of moderate to high methodological quality and were therefore discussed with equal weight. Our narrative synthesis broadly aligns with the core principles of Popay et al. (2006) regarding preliminary synthesis, exploration of relationships, and assessment of robustness. However, we acknowledge that we did not implement the full procedural scope of their guidance, nor did we apply formal evidence grading (e.g., GRADE). Consequently, the conclusions from this section should be considered hypothesis-generating rather than confirmatory.
(8) In addition, the cognitive-set framework proposed in this review still lacks direct empirical validation as an integrated assessment battery. The meta-analytic estimates supporting each module were derived from studies that examined individual modules in isolation, using heterogeneous paradigms and outcome metrics. Whether these four modules, when administered as a coordinated set, yield incremental predictive validity beyond individual modules or alternative combinations remains untested. Future studies employing confirmatory factor analysis, predictive modeling, or intervention designs are needed to establish the framework’s construct and criterion validity.
(9) Considerable heterogeneity existed across three dimensions: the cognitive paradigms used to operationalize ostensibly the same module, the pilot populations sampled, and the flight-performance criteria adopted as outcomes. Regarding paradigm-level variation, working memory was assessed with diverse tasks (*n*-back, complex span, mathematical calculation), and cognitive flexibility through rule-switching or multitasking paradigms, yielding divergent effect sizes (Section 3.3). For population variation, the reviewed studies involved military aviators, commercial airline pilots, general aviation pilots, and cadets, who differ markedly in training, operational context, and selection history; pilot expertise ranged from student pilots to experienced captains, making it uncertain whether observed effects generalize across career stages or whether specific modules are more predictive at particular experience levels. Concerning outcome-measure variation, studies used differing performance criteria (e.g., simulator path deviation, training completion rates, instructor ratings, and operational error rates) reflecting distinct aspects of performance. These three dimensions collectively limit the precision of module-level generalizations, as the meta-analytic estimates in Section 3.3 represent averages across heterogeneous tasks and populations whose relevance to specific domains or pilot groups warrants further exploration.
(10) Conflicting findings were systematically identified and explicitly reported across aviation risk factors. A differentiated approach was adopted for exploring their sources. When inconsistencies could be meaningfully organized along a quantifiable gradient (e.g., altitude levels in hypoxia studies or G-load magnitudes), findings were stratified and interpreted preliminarily. In contrast, when discrepancies arose from methodological heterogeneity at the level of experimental detail, including variations in cognitive paradigms used to index the same construct, differences in participant populations (e.g., cadets versus experienced pilots), or differences in assessment timing (e.g., in-flight versus post-flight), no systematic causal adjudication was attempted. It is acknowledged that not every inconsistency is accompanied by an in-depth causal attribution. This limitation should be considered when interpreting the qualitative synthesis. Future primary research should therefore systematically investigate the sources of heterogeneous findings.

## 5. Conclusions

This review provides the first comprehensive, evidence-based framework that defines the cognitive architecture essential to pilot performance. Synthesizing three decades of empirical research, the findings converge on four interrelated cognitive modules: Perception, Working Memory, Multitasking Flexibility, and Psychomotor. Each represents a distinct but complementary stage in an ongoing cycle of information acquisition, integration, coordination, and execution. Together, they sustain the continuous flow of perception, decision-making, and action that characterizes safe and effective flight.

The same modules that predict successful flight performance also show the highest vulnerability to common aviation stressors such as fatigue, hypoxia, increased workload, gravitational acceleration, and aging. The observed hierarchical pattern of decline indicates that higher-order processes like working memory and flexibility deteriorate before lower-level perceptual or motor functions, offering valuable opportunities for early detection and intervention.

Future research should advance this framework by using longitudinal and real-time designs, integrating physiological and neurocognitive indicators, and applying computational modeling to clarify how these cognitive processes interact and underpin aircraft piloting. Collectively, these efforts can contribute to a more human-centered approach to aviation safety, where cognitive performance is continuously supported, tracked, and optimized.

## Figures and Tables

**Table 1:** Meta-Analytic Associations Between Pilots’ Cognitive Modules and Flight Performance.

Cognitive Module	*k*	*n*	Fisher’s *z*	SE	95% CI	*p*	*r*
Perception	20	16	0.434	0.044	[0.348, 0.519]	<0.001	0.41
Working Memory	19	18	0.424	0.049	[0.328, 0.519]	<0.001	0.40
Flexibility: Multitasking	10	8	0.440	0.059	[0.325, 0.555]	<0.001	0.41
Flexibility: Rule Switching	9	7	0.200	0.056	[0.091, 0.310]	0.0003	0.20
Inhibition	6	6	0.162	0.082	[0.003, 0.322]	0.046	0.16
Spatial Representation	6	6	0.344	0.071	[0.204, 0.483]	<0.001	0.33
Psychomotor	6	6	0.356	0.059	[0.240, 0.473]	<0.001	0.34
Long-Term Memory	8	8	0.255	0.060	[0.137, 0.372]	<0.001	0.25
Problem-Solving	3	3	0.354	0.137	[0.085, 0.622]	0.010	0.34

Note. Fisher’s *z* represents model-estimated mean effect sizes on Fisher’s z scale. *k* = number of effect sizes; *n* = number of independent studies; SE = standard error; CI = confidence interval on the Fisher’s z scale. The back-transformed *r* values provide approximate correlation magnitudes, showing that better cognitive performance is positively associated with flight performance. Model parameters were estimated using restricted maximum likelihood.

**Table 2:** Summary of Study Quality Ratings.

	Factors	High Quality	Moderate Quality	Low Quality	Total
Studies investigating flight performance	-	27	4	0	31
Studies exploring various aviation risk factors	-	63	11	0	74
	Fatigue	12	1	0	13
	Sleep Deprivation	6	1	0	7
	Overload	8	2	0	10
	Stress	6	1	0	7
	Hypoxia & Hypobaria	14	3	0	17
	G-Load	4	1	0	5
	Spatial Disorientation	4	2	0	6
	Aging	14	3	0	17

Note: Study quality was evaluated using the Joanna Briggs Institute (JBI) Critical Appraisal Checklists assessing participant representativeness, experimental validity, reporting completeness, and outcome reliability. Ratings were classified as high, moderate, or low quality. In total, 31 studies correlated pilots’ cognitive performance with flight performance and 74 investigated the effects of aviation-related risk factors on cognitive modules. Because several studies contributed to multiple thematic categories, the cumulative total exceeds the number of studies included in this review (*n* = 93).

**Table 3:** Summary Table of Aviation-Specific Risk Effects.

	Perception	Working Memory	Psychomotor	Flexibility	Long-Term Memory	Inhibition	Problem-Solving	Spatial Represent
Fatigue	*	*m*	*	*	-	-	-	-
Sleep Deprivation	*	*	*	-	-	-	-	-
Overload	*	*	-	*	-	*m*	*	-
Stress	*m*	*m*	-	*m*	-	-	-	-
Hypoxia Hypobaria	*m*	*	-	*	*	*	*	*
G-Load	*	*	*	*	-	*	-	-
Spatial Disorientation	*	*	-	*	-	-	-	-
Aging	*	*	*	*	*	*	*m*	-

Note: “*” indicates consistent and significant evidence of impairment; “*m*” indicates inconsistent findings; “-” indicates limited and no evidence.

## Data Availability

All materials and data generated in the PRISMA workflow are available in the Open Science Framework (OSF) repository. All materials are accessible via the link: [URL https://osf.io/6kw5j, accessed on 2 August 2026].

## Supplementary Materials

The following supporting information can be downloaded at: https://www.mdpi.com/article/10.3390/bs16081348/s1. Table S1. Search Strategies Used for Literature Retrieval Across Databases. Table S2. Inclusion and Exclusion Criteria for Study Selection. Table S3. Model Fit Comparison: Merged Flexibility Model vs. Main Model. Table S4. Merged Flexibility Model: Summary of the Relationship between Pilots’ Cognitive Modules and Flight Performance. Table S5. The Descriptions and Paradigms of Eight Cognitive Modules. Table S6. Joanna Briggs Institute (JBI) Critical Appraisal Checklists Items and Bias Domains. Table S7. Studies Involving Flight Performance and Cognitive Modules. Table S8. Comparison of Multilevel Meta-Analytic Models. Table S9. Estimates From the Additive and Interaction Multilevel Meta-Analytic Models. Table S10. Studies Involving Aviation-Specific Risk Factors and Cognitive Modules. Figure S1. Forest Plot for the Perception Module. Figure S2. Forest Plot for the Working Memory Module. Figure S3. Forest Plot for the Multitasking Flexibility Module. Figure S4. Forest Plot for the Rule-Switching Flexibility Module. Figure S5. Forest Plot for the Inhibition Module. Figure S6. Forest Plot for the Spatial Representation Module. Figure S7. Forest Plot for the Psychomotor Module. Figure S8. Forest Plot for the Long-term Memory Module. Figure S9. Forest Plot for the Problem-Solving Module. Figure S10. Critiplot of Risk-of-Bias Ratings Across All Cognitive Modules. Figure S11. Critiplot of Risk-of-Bias Ratings Across All Aviation-Specific Risk Factors.To maintain conciseness within the main manuscript while facilitating data accessibility, Supplementary Materials have been uploaded to the Open Science Framework (OSF) repository, including the data extraction records, PRISMA checklist, and literature library corresponding to each stage. The OSF repository could be accessed via a link: [URL https://osf.io/6kw5j, accessed on 2 August 2026]. References (Adamson et al., 2010a; Loh et al., 2004; Cak et al., 2020; Cak et al., 2020; Causse et al., 2020; Causse et al., 2011b; Causse et al., 2016; Dehais et al., 2018; Li et al., 2020; Lindseth et al., 2011; Sladky et al., 2016; Wingelaar-Jagt et al., 2023a; Zheng et al., 2023) are cited in the supplementary materials.

## Abbreviations

The following abbreviations are used in this manuscript:

EEG	electroencephalography
fNIRS	functional near-infrared spectroscopy
PVT	Psychomotor Vigilance Task
FAA	Federal Aviation Administration
ICAO	International Civil Aviation Organization
IATA	International Air Transport Association
PRISMA	Preferred Reporting Items for Systematic reviews and Meta-Analyses
G-LOC	G-Induced Loss of Consciousness
+Gz	Gravitational load directed from head to feet
SD	Spatial disorientation
