# Supplementary material for: Cognitive Framework for Aircraft Piloting: A Core Cognition Set
Source: Behav Sci (Basel). 2026 Aug 5;16(8):1348. doi: 10.3390/bs16081348 (PMC13510040; doi:10.3390/bs16081348)
Supplement: Supplementary file 1 [file behavsci-16-01348-s001.zip › behavsci-4334992-supplementary.pdf]

1. Supplementary Tables for Manuscript

Table S1

Search Strategies Used for Literature Retrieval Across Databases

Dataset	Search Strings
PubMed	((aviation) OR (flight) OR (aircraft))AND ((pilot) OR (pilots)OR (aviator)) AND ((cognition) OR (cognitive) OR (working memory) OR (language) OR (executive function) OR (executive functions) OR (memory) OR (problem-solving) OR (mental rotation) OR (perception) OR (psychomotor) OR (Neuropsychological))
Web of Science	(TS=(aviation) OR TS=(flight) OR TS=(aircraft))AND (TS=(pilot) OR TS=(pilots)OR TS=(aviator)) AND (TS=(cognition) OR TS=(cognitive) OR TS=(working memory) OR TS=(language) OR TS=(executive function) OR TS=(executive functions) OR TS=(memory) OR TS=(problem-solving) OR TS=(mental rotation) OR TS=(perception) OR TS=(psychomotor) OR TS=(Neuropsychological))
Scopus	(TITLE-ABS-KEY(aviation) OR TITLE-ABS-KEY(flight) OR TITLE-ABS-KEY(aircraft))AND (TITLE-ABS-KEY(pilot) OR TITLE-ABS-KEY(pilots)OR TITLE-ABS-KEY(aviator)) AND (TITLE-ABS-KEY(cognition) OR TITLE-ABS-KEY(cognitive) OR TITLE-ABS-KEY(working memory) OR TITLE-ABS-KEY(language) OR TITLE-ABS-KEY(executive function) OR TITLE-ABS-KEY(executive functions) OR TITLE-ABS-KEY(memory) OR TITLE-ABS-KEY(problem-solving) OR TITLE-ABS-KEY(mental rotation) OR TITLE-ABS-KEY(perception) OR TITLE-ABS-KEY(psychomotor) OR TITLE-ABS-KEY(Neuropsychological))

Note. Searches were conducted **in PubMed, Web of Science, and Scopus** databases . Boolean operators (AND, OR) were applied to capture studies investigating cognitive functions among pilots or aviators. The search strings included terms related to flight performance, cognition, and major cognitive domains (e.g., working memory, executive function, perception, language, problem solving, and psychomotor ability).

Table S2

Inclusion and Exclusion Criteria for Study Selection

Criterias	Items of Criteria
Inclusion	**1) Focus on pilot performance:** article investigated pilots' cognitive functions and related human factors in aviation operations.
	**2) Availability of full-text English version:** full-text English version of the article could be accessed and reviewed.
	**3) Empirical study design:** article reported original empirical data, including experimental and quasi-experimental designs involving behavioral data collection.
	**4) Report of methodological details:** article provided sufficient information on experimental design, implementation procedures, and data collection methods to allow interpretation and replication.
	**5) Assessment:** Articles that captured quantitative data through behavioral assessments designed to represent participants' cognitive processes, including studies employing established cognitive paradigms or their modified versions, were included.
	**6) Pilot participants:** sample consisted of licensed pilots (Private Pilot, Commercial Pilot, Air Force Pilot, Airline Transport Pilot, Flight Instructor) or recognized pilot cadets currently undergoing flight training.
	**7) Quantitative data report:** article reported quantitative results of cognitive assessments on pilot participants, not merely qualitative or diagnostic descriptions
Exclusion	**1) Topic irrelevance:** article topic is not related to pilots' behavioral performance or cognitive functions, including but not limited to engineering design, algorithm development, chemistry, materials science, biology, medicine, or spaceflight research.
	**2) Unavailable full text in English:** full text or full-text English version of the article could not be obtained.
	**3) Improper study type**: article is clearly not an empirical study, such as a review, meta-analysis, or qualitative study that does not involve behavioral experiments or quantitative data collection.

4) No methodological details: article did not provide sufficient information regarding experimental design, procedures, or data acquisition in the main text.

5) No cognitive assessment: article did not employ cognitive assessment tasks or measures to collect behavioral data from participants.

6) No pilot participants: participants are explicitly reported as non-pilots, or participants are not yet formal pilot cadets under flight training. Articles with unclear participant descriptions were temporarily retained and would be re-examined in the subsequent screening stage.

7) No quantitative data report: article only mentioned cognitive assessments as part of participant grouping, screening, or diagnostic description, without reporting any quantitative cognitive outcomes or statistical results.

Note. Detailed inclusion and exclusion criteria used to identify eligible studies examining pilots' cognitive and behavioral performance. Only empirical, quantitative, and methodologically transparent studies involving licensed pilots or cadets were retained for analysis.

Table S3

Model Fit Comparison: Merged Flexibility Model vs. Main Model

Models	*k*	n	*Q(df)*	*p*	Q_M*(df)*	*p*	AICc	LL	ΔAICc	LRT (χ^2)	LRT *p*
Merged Flexibility model	87	31	180.88 (79)	<.001	196.83 (8)	<.001	-11.68	17.29	-		
Main model	87	31	156.75 (78)	<.001	187.12 (9)	<.001	-19.04	22.28	-7.36	9.98	.0016

***Note*.** The Merged Flexibility model treats rule-switching and multitasking as a signle Flexibility module; the Main Model treats them as separate modules. *k* = number of effect sizes; Q_M = omnibus test of moderators; Q_E = test for residual heterogeneity; AICc = bias-corrected Akaike Information Criterion; LL = log-likelihood; ΔAICc = difference in AICc relative to the merged model (negative values favour the split model); LRT = likelihood-ratio test comparing the two nested models (df = 1).

Table S4

Merged Flexibility Model: Summary of the Relationship between Pilots' Cognitive Modules and Flight Performance

Cognitive Module	*k*	n	Fisher's *z*	SE	95% CI	*p*	*r*
Perception	20	16	.442	.046	[.352, .532]	< .001	.415
Working Memory	19	18	.430	.051	[.330, .530]	< .001	.405
Flexibility	19	15	.301	.046	[.211, .391]	< .001	.293
Inhibition	6	6	.178	.087	[.006, .349]	.042	.176
Spatial Representation	6	6	.334	.078	[.181, .487]	< .001	.322
Psychomotor	6	6	.350	.067	[.219, .480]	< .001	.336
Long-Term Memory	8	8	.254	.065	[.127, .381]	< .001	.249
Problem-Solving	3	3	.356	.142	[.078, .635]	.012	.342

Note. The Merged Flexibility model treats rule-switching and multitasking as a signle Flexibility module. Fisher's *z* values represent weighted mean correlations obtained from a three-level mixed-effects meta-analysis with cognitive modules as fixed moderators and random effects specified at the study and within-study levels. *k* = number of effect sizes; *n* = number of independent studies; SE = standard error; CI = confidence interval. The back-transformed *r* values provide approximate correlation magnitudes, showing that better cognitive performance is positively associated with flight performance.

Table S5

The Descriptions and Paradigms of Eight Cognitive Modules

Cognitive Modules	Descriptions	Paradigms
1) Perception	Involves the recognition, organization, and interpretation of sensory information. Perceptual process facilitates subjects' recognition of certain stimuli (e.g., patterns, sounds, colors, and locations) leading to a binary response regarding the appearance of stimuli (Loh et al., 2004)	Aeromedical Vigilance Test; dichotic lisctning task; Attention Control (MicroCog); Attribution Identification (Cogscreen); Choice Reaction Time (Cogscreen)(MicroCog); Trail Making Test Part A; duration discrimination task; dichotic lisctning task; Psychomotor Vigilance Test; Processing speed (Cogscreen) (Wiener); signal detection (Vienna Test System); Symbol Digit Coding (Cogscreen; WAIS-IV); Target Hitting Test; target stimulus discrimination; Tone discriminating task; Visual Scanning (Cogscreen)
2) Working Memory	Can be conceptualized as the brain's 'workspace,' where information is actively processed and manipulated to facilitate complex cognitive tasks such as learning, reasoning, and comprehension (Baddeley, 1992). Typically, presents a continuous stream of stimuli, requiring individuals to continuously store and process these stimuli to execute specified responses.	Corsi block-tapping test; Computational task; Digit Span task; N-back; N-back (auditory); Dual-N-back; Elevator test; Letter-number sequencing (WAIS); Paced Auditory Serial Addition Test; Auditory sequence comparison (Cogscreen)
3) Cognitive Flexibility	Refer to switching abstract mental frameworks to address diverse tasks. Enable individuals to appropriately respond to changing environments or multitasking, instead of being trapped by specific behavioral or cognitive patterns (Scott, 1962; Ionescu, 2012). Typically requires individuals to switch between multiple tasks or rules. Lower switching costs correspond to stronger cognitive flexibility.	Color trail test 2; Divided attention (Cogscreen); Dual task performance; Dual-N-back; Dual-Task Costs; executive function (Cogscreen); Modified Five-Point Task; Multitasking Performance; Pathfinder (Cogscreen); Shifting attention (Cogscreen); Switching of Attention (SWATT); Trail Making Test Part B; Wisconsin Card Sorting Task
4) Cognitive Inhibition	Involve resistance to distracting perceptions and impulses, for directing attention towards goal-directed information and actions (Harnishfeger, 1995). Typically present the stimuli containing incongruent or congruent features, requiring individuals to ignore non-target feature and merely respond to target features.	Antisaccade Task; Attention Network Test; Stop-change task; double-choice Oddball task; Eriksen Flanker; Go /No Go; Stroop (auditory); Stroop (emotion); Stroop (spatial); Stroop (volume);
5) Spatial Representation	Involves the process where individuals mentally visualize and manipulate objects, specifically the capacity to generate mental images of 2-dimensional or 3-dimensional objects from varying angles or perspectives.	Completion of Rotated Figures Test; Directional Judgment Task; Manikin (Cogscreen); Map Description Task; Mental Rotation Task; Shepard and Metzler Task; Silcton environment task; spatial orientation tasks (Planikin); Spatial Processing (MicroCog); Three-Dimensional (3D) Rotation Task; Vandenberg's mental rotation test; Walking Corsi Test; Way-Figures Test
6) Psychomotor	Entail the transformation of cerebral activity into physical movements (Lezak et al., 2012), focusing on hand-eye coordination, manual dexterity, and fine motor precision.	Complex Coordination Test; Grooved Pegboard; motor coordination (Cogscreen); Piórkowski Apparatus; psychomotor test (Wiener); Tapping Test of Fepsy; tracking task (Cogscreen)
7) Long-Term Memory	Encompass the processes of storing, encoding, and retrieving information across prolonged durations. In contrast to working memory, assessments entail longer intervals between encoding and retrieval, diverging from the focus on immediate memory processing within seconds. Memorizing process can be categorized into various types, such as procedural memory, semantic memory, and episodic memory etc.	Amsterdam Short-term Memory Test; Delayed Matching-to-Sample Test; California Verbal Learning Test-II; Aviation Prospective memory Task; learning and delayed recall; Matching (Cogscreen); Memory task (MicroCog) (ANAM4)(WMS-IV); Rey Learning Test; Rey-Osterrieth Complex Figure; visual associative memory (Cogscreen)

8) Problem-solving	Encompasses the analysis of goal-directed information and the structuring of thoughts beyond immediate responses, ultimately outputting guidance for subsequent actions. Relevant cognitive paradigms introduce diverse puzzle formats (e.g., planning and reasoning), tasking participants to provide a solution as soon as possible.	Logical relations test (ANAM4); One Touch Stockings; Picture Arrangement (WAIS-III); reasoning test (MicroCog); Test of Nonverbal Intelligence; Tower puzzle test; Visual Puzzles of (WAIS-IV)

Table S6

Joanna Briggs Institute (JBI) Critical Appraisal Checklists Items and Bias Domines

Study type	Item	Bias domine	Item code
Randomized Controlled			
	1. Was true randomization used for assignment of participants to treatment groups?	Selection	RCT_1
	2. Was allocation to groups concealed?	Selection	RCT_2
	3. Were treatment groups similar at the baseline?	Selection	RCT_3
	4. Were participants blind to treatment assignment?	Performance	RCT_4
	5. Were those delivering treatment blind to treatment assignment?	Performance	RCT_5
	6. Were outcomes assessors blind to treatment assignment?	Detection	RCT_6
	7. Were treatment groups treated identically other than the intervention of interest?	Performance	RCT_7
	8. Was follow up complete and if not, were differences between groups in terms of their follow up adequately described and analyzed?	Attrition	RCT_8
	9. Were participants analyzed in the groups to which they were randomized?	Attrition	RCT_9
	10. Were outcomes measured in the same way for treatment groups?	Detection	RCT_10
	11. Were outcomes measured in a reliable way?	Detection	RCT_11
	12. Was appropriate statistical analysis used?	Reporting	RCT_12
	13. Was the trial design appropriate for the topic, and any deviations from the standard RCT design accounted for in the conduct and analysis?	Reporting	RCT_13
Quasi Experimental			
	1. Is it clear in the study what is the 'cause' and what is the 'effect'?	Selection	QE_1
	2. Were the participants included in any comparisons similar?	Selection	QE_2
	3. Were the participants included in any comparisons receiving similar treatment/care, other than the exposure or intervention of interest?	Performance	QE_3
	4. Was there a control group?	Selection	QE_4
	5. Were there multiple measurements of the outcome both pre and post the intervention /exposure?	Detection	QE_5
	6. Was follow up complete and if not, were differences between groups in terms of their follow up adequately described and analyzed?	Attrition	QE_6
	7. Were the outcomes of participants included in any comparisons measured in the same way?	Detection	QE_7
	8. Were outcomes measured in a reliable way?	Detection	QE_8
	9. Was appropriate statistical analysis used?	Reporting	QE_9
Cross-Sectional			
	1. Were the criteria for inclusion in the sample clearly defined?	Selection	CS_1
	2. Were the study subjects and the setting described in detail?	Selection	CS_2
	3. Was the exposure measured in a valid and reliable way?	Detection	CS_3
	4. Were objective, standard criteria used for measurement of the condition?	Detection	CS_4
	5. Were confounding factors identified?	Confounding	CS_5

	6. Were strategies to deal with confounding factors stated?	Confounding	CS_6
	7. Were the outcomes measured in a valid and reliable way?	Detection	CS_7
	8. Was appropriate statistical analysis used?	Reporting	CS_8
Cohort			
	1. Were the two groups similar and recruited from the same population?	Selection	C_1
	2. Were the exposures measured similarly to assign people to both exposed and unexposed groups?	Detection	C_2
	3. Was the exposure measured in a valid and reliable way?	Detection	C_3
	4. Were confounding factors identified?	Confounding	C_4
	5. Were strategies to deal with confounding factors stated?	Confounding	C_5
	6. Were the groups/participants free of the outcome at the start of the study (or at the moment of exposure)?	Selection	C_6
	7. Were the outcomes measured in a valid and reliable way?	Detection	C_7
	8. Was the follow up time reported and sufficient to be long enough for outcomes to occur?	Attrition	C_8
	9. Was follow up complete, and if not, were the reasons to loss to follow up described and explored?	Attrition	C_9
	10. Were strategies to address incomplete follow up utilized?	Attrition	C_10
	11. Was appropriate statistical analysis used?	Reporting	C_11

Note. Checklist items from the *Joanna Briggs Institute (JBI) Critical Appraisal Tools* were used to classify potential bias sources in different studies across six domains: selection, performance, detection, attrition, confounding, and reporting bias. The item codes were used to record during data extraction. Reviewers rated each applicable item as “YES,” “NO,” “UNCLEAR,” or “NOT APPLICABLE.”

Table S7

Studies Involving Flight Performance and Cognitive Modules

	Study ID	Sample size	Pilot Type
Flight Performance			
	(Causse et al., 2011a)	32	Private Pilots
	(Taylor et al., 2000)	100	Civilian Pilots
	(Causse et al., 2011b)	24	Private Pilots
	(Lopez et al., 2012)	10	Military Pilots
	(Yesavage et al., 2011)	276	Civilian Pilots
	(Adamson et al., 2010b)	50	General Aviation Pilots
	(Ayala et al., 2024)	22	Cadet Pilots
	(Cak et al., 2020)	36	Unspecified
	(Griffin, 1998)	452	Cadet Pilots
	(Guan, 2003)	122	Cadet Pilots
	(Kennedy et al., 2013)	236	Unspecified
	(Kennedy et al., 2010)	72	Civilian Pilots
	(Kennedy et al., 2015)	263	Civilian Pilots
	(Li et al., 2020)	30	Military Pilots
	(O'Hagan et al., 2020)	5	Airline Pilots
	(Van Benthem and Herdman, 2021b)	36	Unspecified
	(Van Benthem and Herdman, 2021a)	51	Unspecified
	(Leino et al., 1999)	35	Cadet Pilots
	(Bouak et al., 2018)	16	Military Pilots
	(Taylor et al., 2011)	139	Airline Pilots
	(Sohn and Doane, 2004)	52	Airline Pilots
	(Van Benthem and Herdman, 2020)	112	Unspecified
	(Causse et al., 2017)	26	Cadet Pilots
	(Bednarek et al., 2019)	66	Military Pilots
	(Caldwell et al., 2004)	10	Military Pilots
	(Causse et al., 2020)	18	Airline Pilots
	(Lindseth et al., 2011)	45	Cadet Pilots
	(Morrow et al., 2003)	91	Airline Pilots
	(Zheng et al., 2023)	40	Cadet Pilots/Unspecified
	(Xie et al., 2024)	56	Cadet Pilots
	(Sladky et al., 2016)	18	Airline Pilots

Table S8

Comparison of Multilevel Meta-Analytic Models

Models	$\tau^2 1$	$\tau^2 2$	*Q(df)*	*p*	Q_M*(df)*	*p*	R^2 %	AICc	LL	ΔAICc	LRT (χ^2)	LRT *p*
Null	.0050	.0154	209.97 (86)	<.001			-	-12.52	9.40	-		
Main	.0089	.0072	156.75 (78)	<.001	187.12 (9)	<.001	21.08	-19.04	22.28	-6.52	25.75 (8)	.0012
Additive	.0105	.0068	151.87 (76)	<.001	31.05 (10)	<.001	15.20	−15.34	23.16	+3.70	1.77 (2)	.4135
Interaction	.0079	.0074	129.02 (65)	<.001	45.12 (21)	.0017	25.00	3.90	22.28	+19.23	17.13 (11)	.1040

***Note*.** The null model contained no moderators; the main model included cognitive module as the sole moderator; the additive model included cognitive module and flight-measure type as additive moderators; and the interaction model included the main effects of cognitive module and flight-measure type and their interaction. The main model was the prespecified primary model, the additive model was fitted as a sensitivity analysis, and the interaction model was fitted as an exploratory moderator analysis. A total of k = 87 effect sizes from n = 31 independent studies were included. $\tau^2 1$ = between-study variance component (*Study_ID_Merged*); $\tau^2 2$ = within-study/effect-level variance component (*EffectID_global* nested within studies); *Q*(df)= Cochran's Q test for total heterogeneity in the null model, Cochran's Q test for residual heterogeneity after accounting for moderators in the full model; Q_M *(df)* = omnibus test for the moderator (Cognitive Modules), examining whether the mean effect sizes differ significantly across cognitive modules; R^2 = proportion of total variance explained by the moderator, calculated as $\left(\tau^2_{\text{total, Null}} - \tau^2_{\text{total, Full}}\right)/\tau^2_{\text{total, Null}}$. All variance components are reported on the Fisher's Z scale; AICc = Akaike Information Criterion corrected for small sample sizes; LL = log-likelihood; LRT = likelihood-ratio test comparing the two nested models.

Table S9

Estimates From the Additive and Interaction Multilevel Meta-Analytic Models

Model	Predictor	*b*	SE	95% CI	*z*	*p*
Additive	***Intercept***	.437	.050	[.339, .536]	8.692	< .001
	Cognitive module					
	Working Memory	−.008	0.062	[−.129, .113]	−0.131	0.896
	Flexibility: Multitasking	0.013	0.065	[−.115, .141]	0.196	0.845
	Flexibility: Rule Switching	−.231	0.061	[−.351, −.111]	−3.776	< .001
	Inhibition	−.281	0.092	[−.461, −.102]	−3.069	0.002
	Spatial Representation	−.093	0.08	[−.251, .064]	−1.161	0.246
	Psychomotor	−.078	0.063	[−.200, .045]	−1.244	0.213
	Long-Term Memory	−.190	0.07	[−.326, −.053]	−2.720	0.007
	Problem-Solving	−.045	0.146	[−.331, .240]	−0.310	0.756
	Flight measure type					
	Flight Control	−.068	0.08	[−.224, .088]	−0.855	0.393
	Situation Awareness	0.039	0.073	[−.103, .182]	0.542	0.588
Interaction	***Intercept***	0.444	0.05	[.346, .543]	8.83	< .001
	Cognitive module					
	Working Memory	−.106	0.084	[−.271, .060]	−1.251	0.211
	Flexibility: Multitasking	0.037	0.078	[−.116, .191]	0.476	0.634
	Flexibility: Rule Switching	−.217	0.068	[−.351, −.083]	−3.179	0.002
	Inhibition	0.018	0.205	[−.384, .420]	0.088	0.93
	Spatial Representation	−.129	0.109	[−.343, .085]	−1.184	0.236
	Psychomotor	−.085	0.065	[−.213, .042]	−1.311	0.19
	Long-Term Memory	−.239	0.078	[−.391, −.088]	−3.090	0.002
	Problem-Solving	−.076	0.175	[−.419, .267]	−0.436	0.663
	Flight measure type					
	Flight Control	−.065	0.134	[−.327, .197]	−0.484	0.628
	Situation Awareness	−.118	0.147	[−.406, .171]	−0.798	0.425
	Cognitive Module × Flight Control					
	Working Memory × Flight Control	0.095	0.167	[−.233, .423]	0.568	0.57
	Flexibility: Multitasking × Flight Control	0.045	0.191	[−.329, .419]	0.234	0.815
	Flexibility: Rule Switching × Flight Control	−.129	0.185	[−.492, .234]	−0.698	0.485
	Inhibition × Flight Control	−.440	0.284	[−.995, .116]	−1.552	0.121
	Spatial Representation × Flight Control	0.117	0.257	[−.386, .620]	0.456	0.649
	Psychomotor × Flight Control	—	—	—	—	—
	Long-Term Memory × Flight Control	—	—	—	—	—

Problem-Solving × Flight Control	—	—	—	—	—
Cognitive Module × Situation Awareness					
Working Memory × Situation Awareness	0.352	0.18	[−.001, .705]	1.957	0.0503
Flexibility: Multitasking × Situation Awareness	−.203	0.208	[−.611, .204]	−0.978	0.328
Flexibility: Rule Switching × Situation Awareness	0.213	0.317	[−.408, .835]	0.673	0.501
Inhibition × Situation Awareness	−.161	0.266	[−.682, .360]	−0.605	0.545
Spatial Representation × Situation Awareness	0.183	0.206	[−.221, .586]	0.887	0.375
Psychomotor × Situation Awareness	—	—	—	—	—
Long-Term Memory × Situation Awareness	0.316	0.199	[−.073, .705]	1.593	0.111
Problem-Solving × Situation Awareness	—	—	—	—	—

***Note*.** The additive model included cognitive module and flight-measure type as additive moderators, whereas the interaction model additionally included their interaction. *Perception* and *composite_performance* were the reference categories for cognitive module and flight-measure type, respectively; thus, the intercept represents the estimated mean Fisher's z for Perception and composite performance. In the additive model, coefficients represent adjusted contrasts with the corresponding reference categories. In the interaction model, cognitive-module and flight-measure-type coefficients represent conditional contrasts at composite performance and Perception, respectively, whereas interaction coefficients represent differences in cognitive-module contrasts relative to composite performance. b = unstandardized meta-regression coefficient on the Fisher's z scale; SE = standard error; CI = confidence interval; z = Wald test statistic. Models were estimated using restricted maximum likelihood. Dashes indicate nonestimable coefficients due to redundant predictors. Omnibus tests and model-fit comparisons are reported in Table S8.

Table S10

Studies Involving Aviation-Specific Risk Factors and Cognitive Modules

Themes	Study ID	Sample size	Pilot Type
Fatigue			
	(Rosa et al., 2020)	39	Military Pilots
	(Arsintescu et al., 2022)	90	Airline Pilots
	(Bartulovic et al., 2023)	4	Airline Pilots
	(da Silva et al., 2021)	3	Military Pilots
	(Fletcher et al., 2022)	162	Military Pilots
	(Gander et al., 2014)	144	Airline Pilots
	(Gander et al., 2013)	70	Airline Pilots
	(Gordon et al., 2016)	39	Cadet Pilots
	(Li et al., 2020)	71	Airline Pilots
	(Roach et al., 2012)	19	Airline Pilots
	(McMahon and Newman, 2018)	10	Helicopter Pilots
	(Rosa et al., 2020)	39	Military Pilots
	(Gordon et al., 2016)	39	Cadet Pilots
	(Rabinowitz et al., 2009)	63	Military Pilots
Sleep Derivation			
	(Lopez et al., 2012)	10	Military Pilots
	(Algranati et al., 2024)	179	Military Pilots
	(O'Hagan et al., 2020)	5	Airline Pilots
	(Russo et al., 2004)	8	Military Pilots
	(Wingelaar-Jagt et al., 2023b)	30	Military Pilots
	(Wingelaar-Jagt et al., 2023a)	32	Military Pilots
	(Caldwell et al., 2004)	10	Military Pilots
Workload			
	(Ayala et al., 2024)	22	Cadet Pilots
	(Dehais et al., 2019)	13	Military Pilots
	(Galant-Gołębiewska et al., 2020)	3	Cadet Pilots
	(Peng et al., 2022)	24	Cadet Pilots
	(Williams, 1995)	48	Cadet Pilots
	(Causse et al., 2024)	20	Private Pilots
	(Zakay and Shub, 1998)	61	Unspecified
	(Causse et al., 2017)	26	Cadet Pilots
	(Causse et al., 2016)	24	Cadet Pilots
	(Hernandez-Sabate et al., 2022)	21	Unspecified
Stress			
	(Leino et al., 1999)	35	Cadet Pilots
	(Liang et al., 2024)	42	Unspecified
	(Deniel et al., 2023)	6	Cadet Pilots
	(Combs et al., 2021)	17	Military Pilots
	(Pattyn et al., 2014)	12	Cadet Pilots
	(Causse et al., 2024)	20	Private Pilots
Hypoxia/Hypobaria			

	(Asmaro et al., 2013)	29	Unspecified
	(Bustamante-Sanchez et al., 2019)	23	Military Pilots
	(McGuire et al., 2014)	106	Military Pilots
	(Nation et al., 2017)	11	Military Pilots
	(Takács et al., 2017)	25	Military Pilots
	(Vacchiano et al., 2008)	74	Military Pilots
	(Bouak et al., 2018)	16	Military Pilots
	(Bouak et al., 2019)	17	Military Pilots
	(Peacock et al., 2017)	10	Unspecified
	(Legg et al., 2016)	36	Military Pilots
	(Aebi et al., 2020)	16	Cadet Pilots
	(Ilbasmis, 2024)	25	Cadet Pilots
	(Malle et al., 2013)	57	Military Pilots
	(Wen et al., 2020)	66	Military Pilots
	(Thropp and Buza, 2019)	24	Unspecified
	(McGuire et al., 2016)	106	Military Pilots
G-Load			
	(Biernacki et al., 2013)	20	Cadet Pilots
	(Ercan and Gunduz, 2020)	29	Unspecified
	(Truszczynski et al., 2014)	40	Military Pilots
	(Tripp et al., 2006)	13	Airline Pilots
	(Dalecki et al., 2010)	10	Military Pilots
Spatial Disorientation			
	(Stróżak et al., 2018)	32	Military Pilots
	(Balaj et al., 2019)	20	Unspecified
	(Lewkowicz et al., 2018)	40	Military Pilots
	(Bednarek et al., 2019)	66	Military Pilots
	(Lewkowicz et al., 2019)	39	Military Pilots
	(Tornero Aguilera et al., 2022)	25	Military Pilots
	(Webb et al., 2012)	36	Cadet Pilots
Aging			
	(Hardy et al., 2007)	220	Airline Pilots
	(Causse et al., 2011a)	32	Private Pilots
	(Taylor et al., 2000)	100	Civilian Pilots
	(Yesavage et al., 2011)	276	Civilian Pilots
	(Adamson et al., 2010a)	51	General Aviation Pilots
	(Arikan et al., 2018)	147	Airline Pilots
	(Kennedy et al., 2013)	236	Unspecified
	(Kennedy et al., 2010)	72	Civilian Pilots
	(Kennedy et al., 2015)	263	Civilian Pilots
	(Morrow et al., 2003)	91	Airline Pilots
	(Taylor et al., 2011)	139	Airline Pilots
	(Tsang, 1998)	45	Unspecified
	(Adamson et al., 2010b)	50	General Aviation Pilots

(Audu et al., 2021)	47	Unspecified
(Van Benthem and Herdman, 2020)	112	Unspecified
(Causse et al., 2019)	61	Private Pilots

2. Supplementary Figures for Manuscript

Figure S1

Forest Plot for the Perception Module

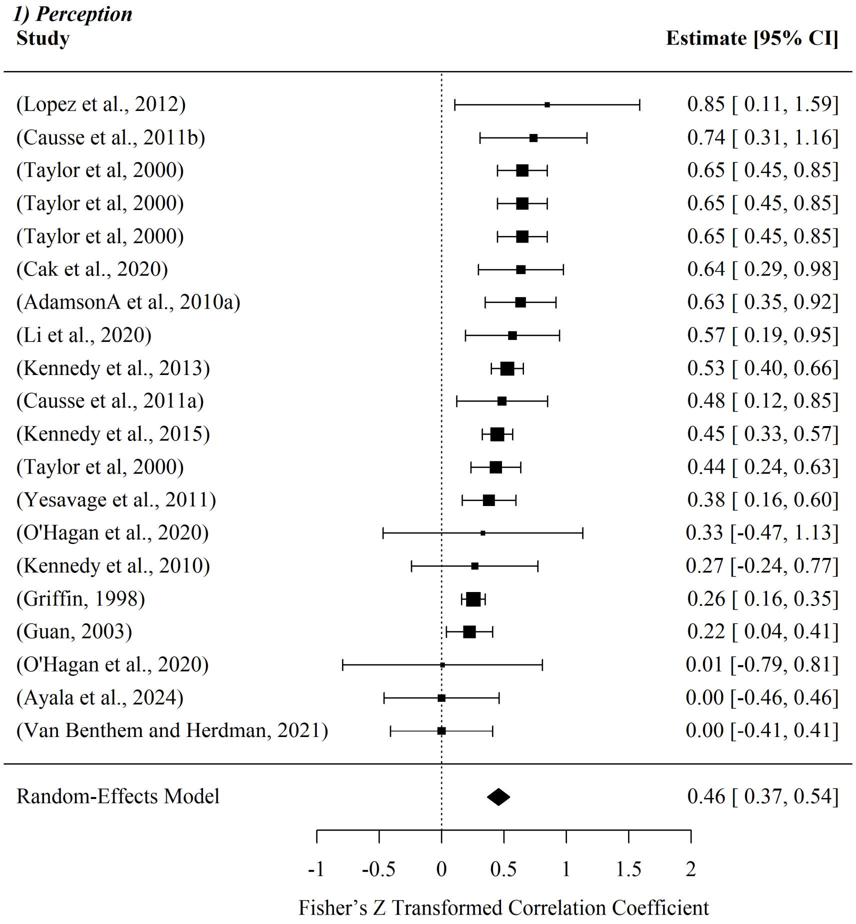

Note. This forest plot presents the Fisher's z-transformed correlation coefficients and corresponding 95% confidence intervals for studies examining the association between pilots' perception module and flight performance. Each square represents the effect size of an individual study, with the size of the square proportional to its weight in the meta-analysis. The diamond at the bottom denotes the pooled random-effects estimate ($z = 0.46$ [95% CI 0.37, 0.54]), indicating a moderate positive relationship between perception and flight performance.

Figure S2

Forest Plot for the Working Memory Module

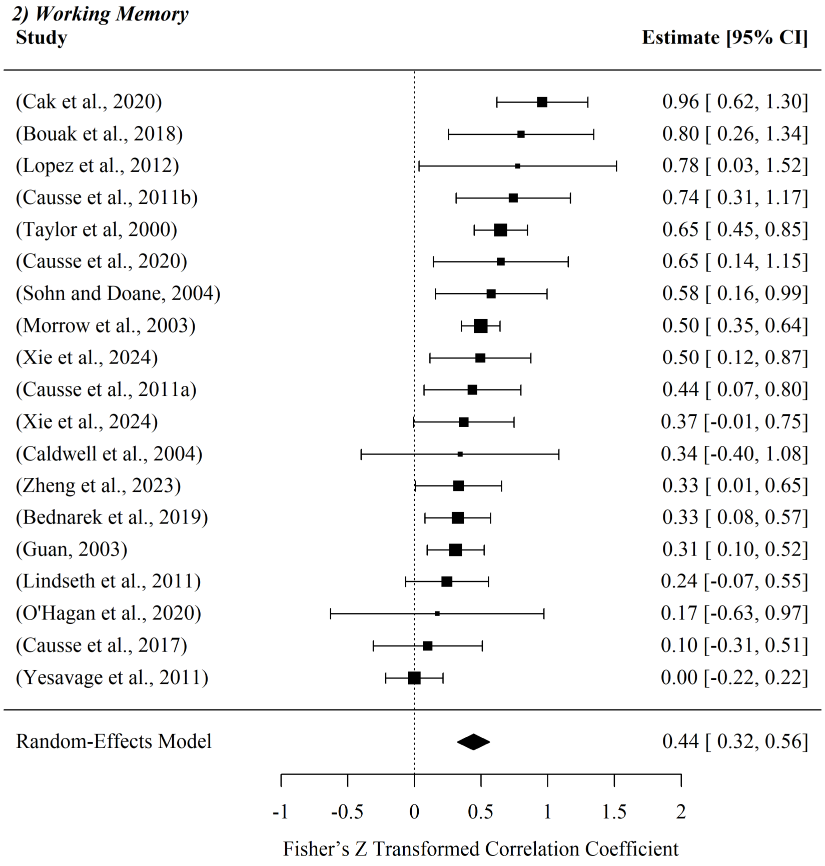

Note. This forest plot presents the Fisher's z-transformed correlation coefficients and corresponding 95% confidence intervals for studies examining the association between pilots' working memory module and flight performance. Each square represents the effect size of an individual study, with the size of the square proportional to its weight in the meta-analysis. The diamond at the bottom denotes the pooled random-effects estimate ($z = 0.44$ [95% CI 0.32, 0.56]), indicating a moderate positive relationship between perception and flight performance.

Figure S3

Forest Plot for the Multitasking Flexibility Module

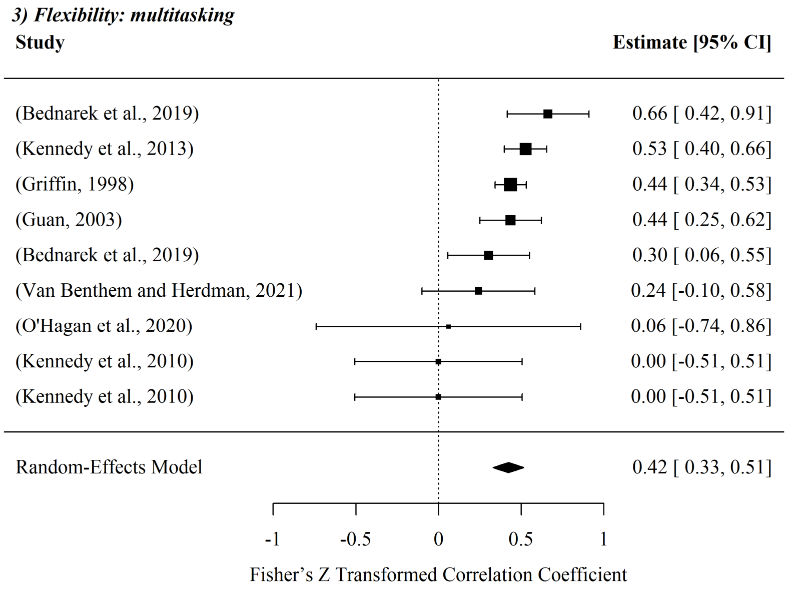

Note. This forest plot presents the Fisher's z-transformed correlation coefficients and corresponding 95% confidence intervals for studies examining the association between pilots' multitasking flexibility module and flight performance. Each square represents the effect size of an individual study, with the size of the square proportional to its weight in the meta-analysis. The diamond at the bottom denotes the pooled random-effects estimate ($z = 0.42$ [95% CI 0.33, 0.51]), indicating a moderate positive relationship between perception and flight performance.

Figure S4

Forest Plot for the Rule-Switching Flexibility Module

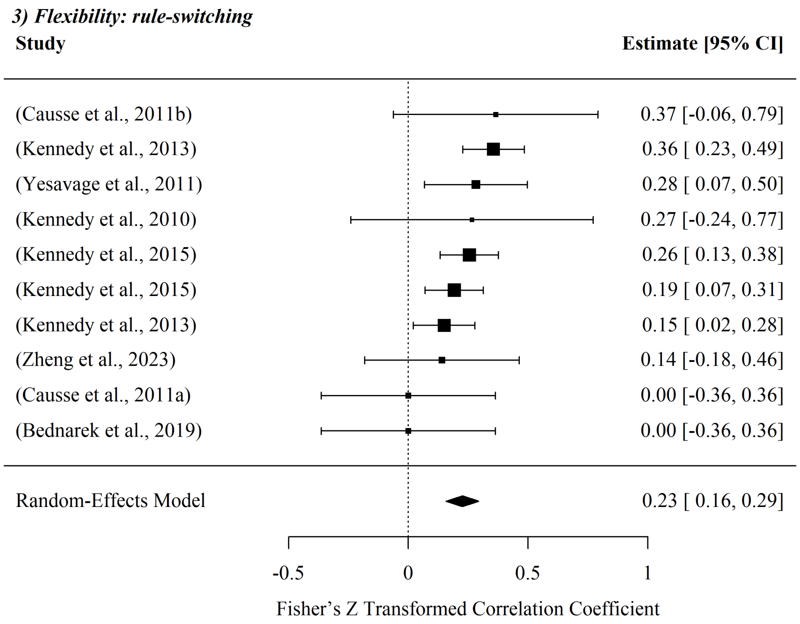

Note. This forest plot presents the Fisher's z-transformed correlation coefficients and corresponding 95% confidence intervals for studies examining the association between pilots' rule-switching flexibility module and flight performance. Each square represents the effect size of an individual study, with the size of the square proportional to its weight in the meta-analysis. The diamond at the bottom denotes the pooled random-effects estimate ($z = 0.23$ [95% CI 0.16, 0.29]), indicating a moderate positive relationship between perception and flight performance.

Figure S5

Forest Plot for the Inhibition Module

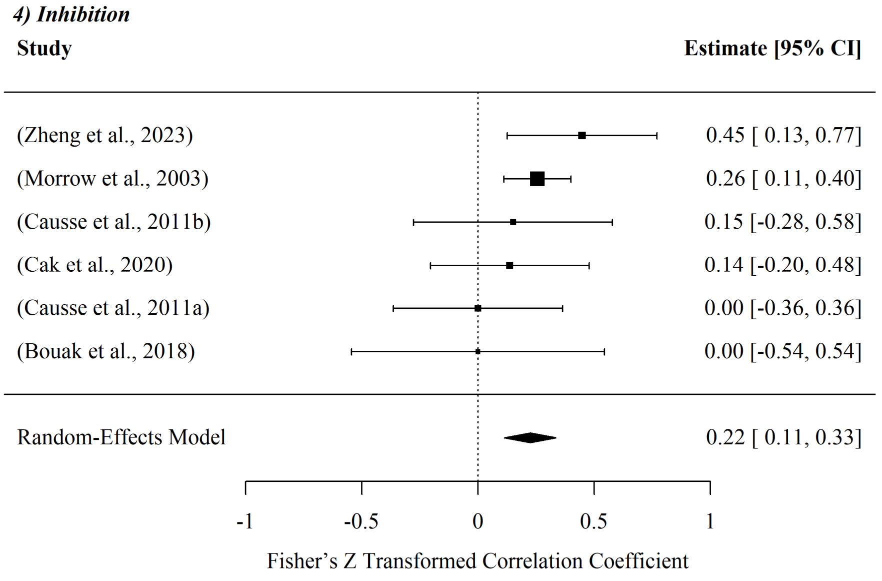

Note. This forest plot presents the Fisher's z-transformed correlation coefficients and corresponding 95% confidence intervals for studies examining the association between pilots' inhibition module and flight performance. Each square represents the effect size of an individual study, with the size of the square proportional to its weight in the meta-analysis. The diamond at the bottom denotes the pooled random-effects estimate ($z = 0.22$ [95% CI 0.11, 0.33]), indicating a moderate positive relationship between perception and flight performance.

Figure S6

Forest Plot for the Spatial Representation Module

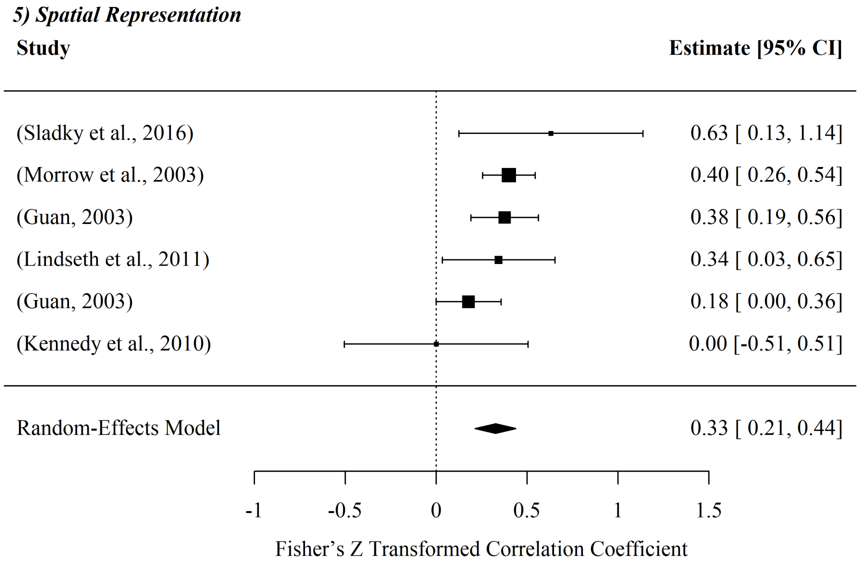

Note. This forest plot presents the Fisher's z-transformed correlation coefficients and corresponding 95% confidence intervals for studies examining the association between pilots' spatial representation module and flight performance. Each square represents the effect size of an individual study, with the size of the square proportional to its weight in the meta-analysis. The diamond at the bottom denotes the pooled random-effects estimate ($z = 0.33$ [95% CI 0.21, 0.44]), indicating a moderate positive relationship between perception and flight performance.

Figure S7

Forest Plot for the Psychomotor Module

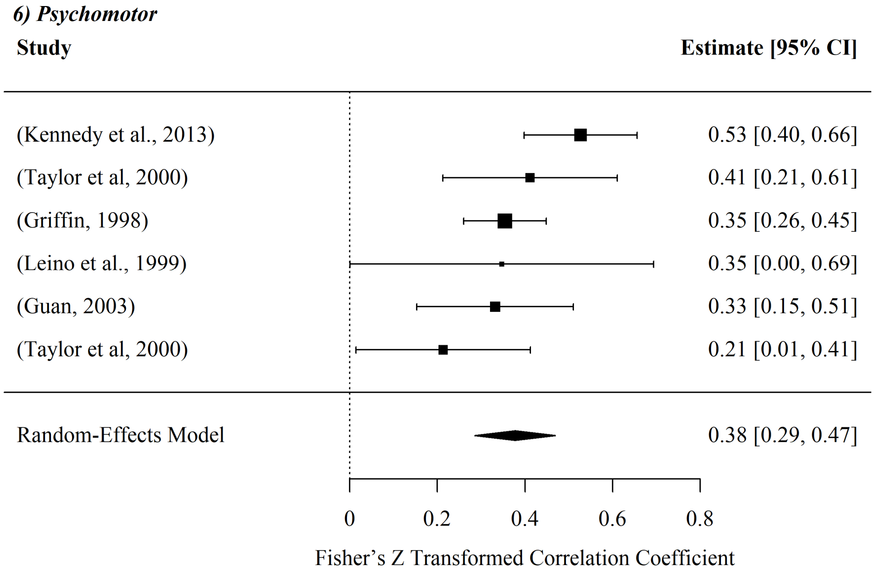

Note. This forest plot presents the Fisher's z-transformed correlation coefficients and corresponding 95% confidence intervals for studies examining the association between pilots' psychomotor module and flight performance. Each square represents the effect size of an individual study, with the size of the square proportional to its weight in the meta-analysis. The diamond at the bottom denotes the pooled random-effects estimate ($z = 0.38$ [95% CI 0.29, 0.47]), indicating a moderate positive relationship between perception and flight performance.

Figure S8

Forest Plot for the Long-term Memory Module

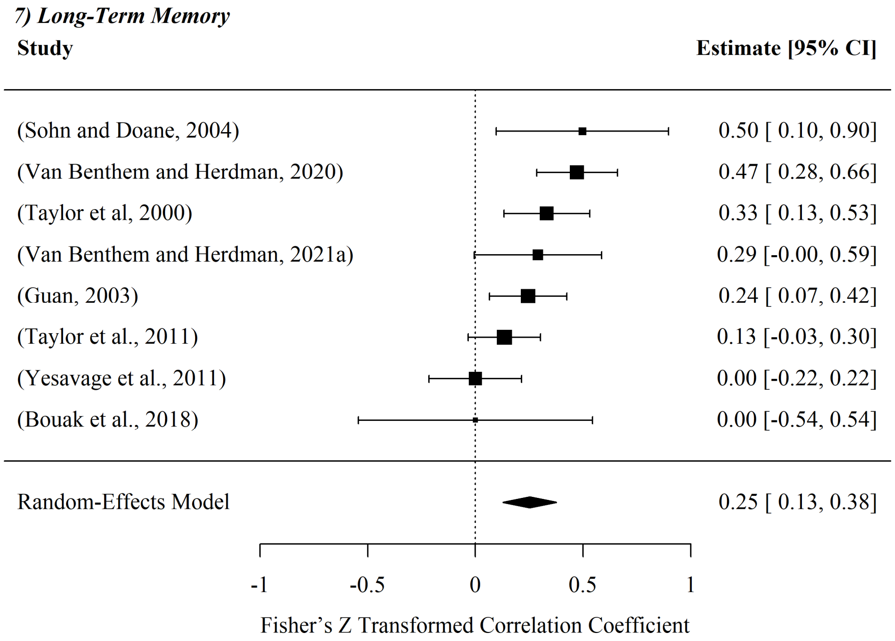

Note. This forest plot presents the Fisher's z-transformed correlation coefficients and corresponding 95% confidence intervals for studies examining the association between pilots' long-term memory module and flight performance. Each square represents the effect size of an individual study, with the size of the square proportional to its weight in the meta-analysis. The diamond at the bottom denotes the pooled random-effects estimate ($z = 0.25$ [95% CI 0.13, 0.38]), indicating a moderate positive relationship between perception and flight performance.

Figure S9

Forest Plot for the Problem-Solving Module

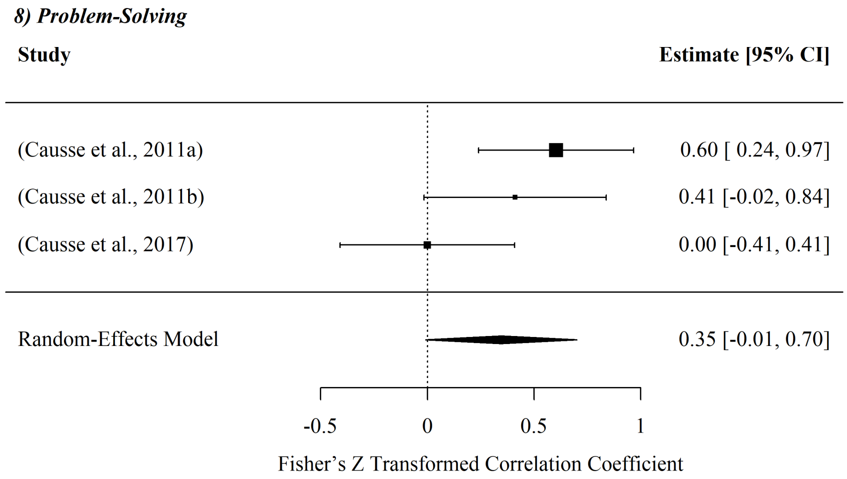

Note. This forest plot presents the Fisher's z-transformed correlation coefficients and corresponding 95% confidence intervals for studies examining the association between pilots' problem-solving module and flight performance. Each square represents the effect size of an individual study, with the size of the square proportional to its weight in the meta-analysis. The diamond at the bottom denotes the pooled random-effects estimate ($z = 0.35$ [95% CI -0.01, 0.70]), indicating a moderate positive relationship between perception and flight performance.

Figure S10

Critiplot of Risk-of-Bias Ratings Across All Cognitive Modules

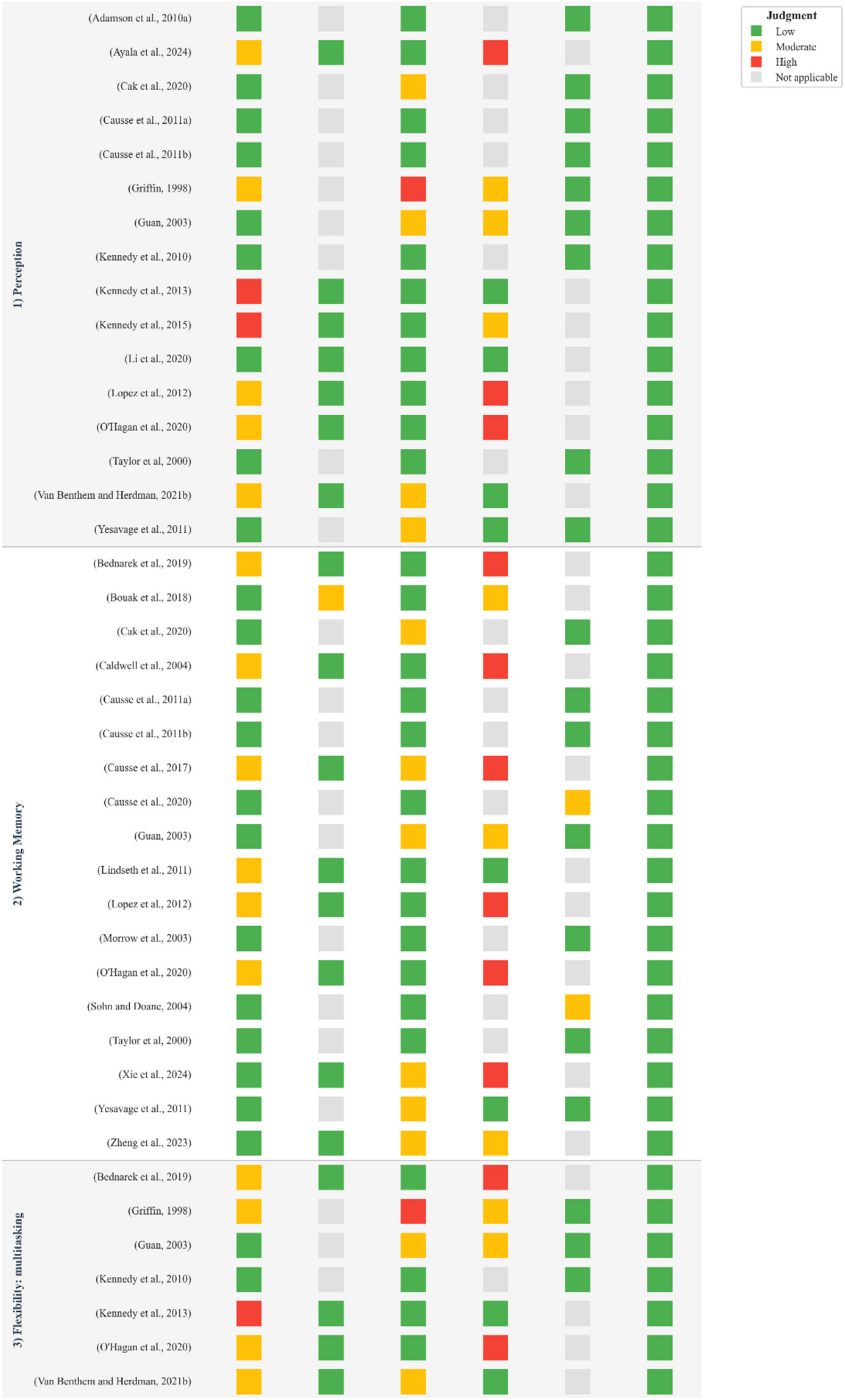

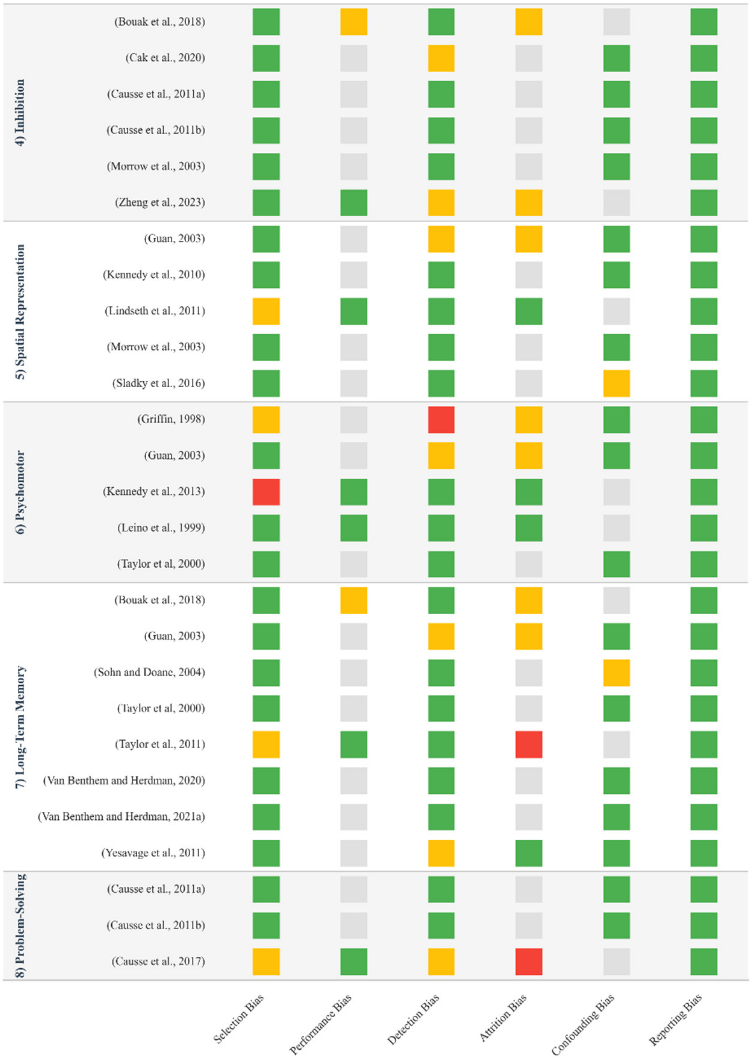

Note. This figure summarizes the risk-of-bias ratings for the 31 studies that investigated the association between pilots' cognitive modules and flight performance. Each study was evaluated across six bias domains (selection, performance, detection, attrition, confounding, and reporting) using the Joanna Briggs Institute (JBI) Critical Appraisal Checklists. Studies are grouped by cognitive module, with colored squares representing the reviewers' judgments (green = low, yellow = moderate, red = high, gray = not applicable). Two independent reviewers assessed methodological quality, and discrepancies were resolved through discussion.

Figure S11

Critiplot of Risk-of-Bias Ratings Across All Aviation-Specific Risk Factors

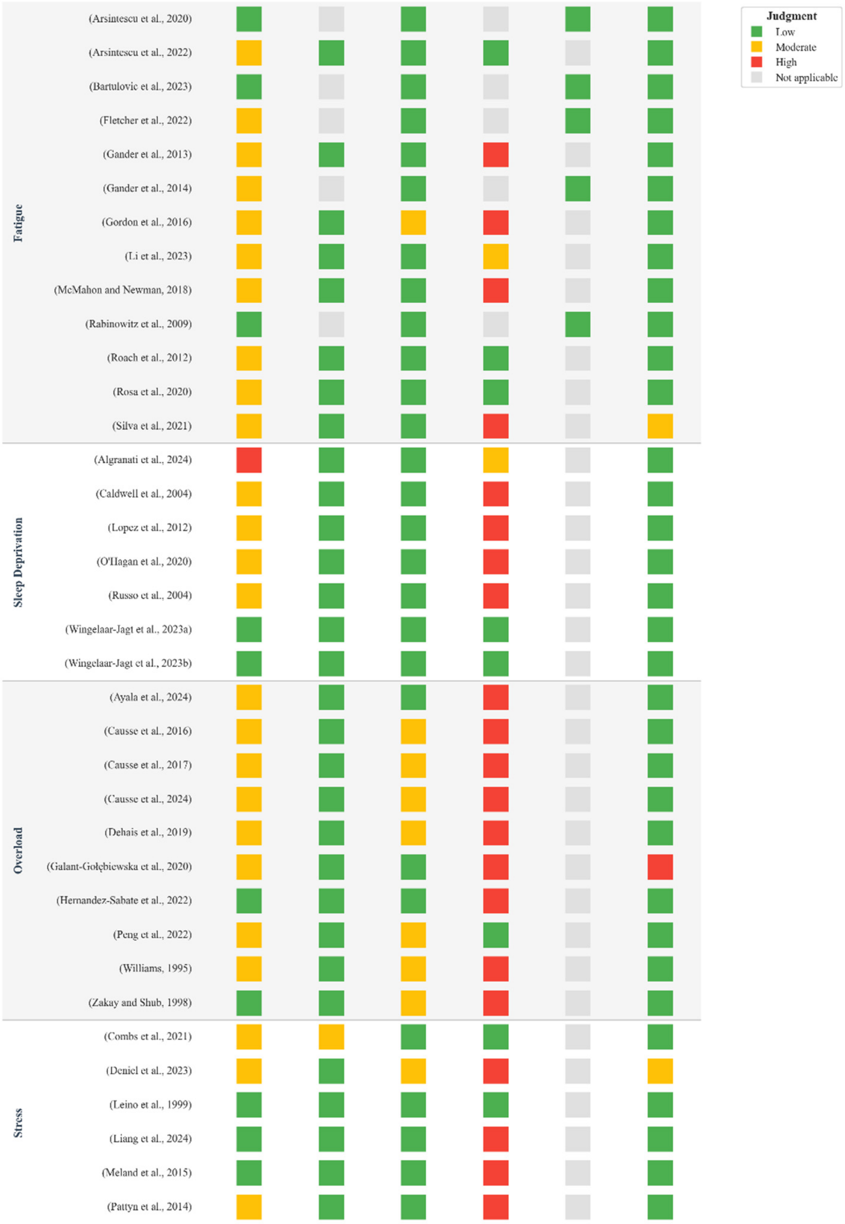

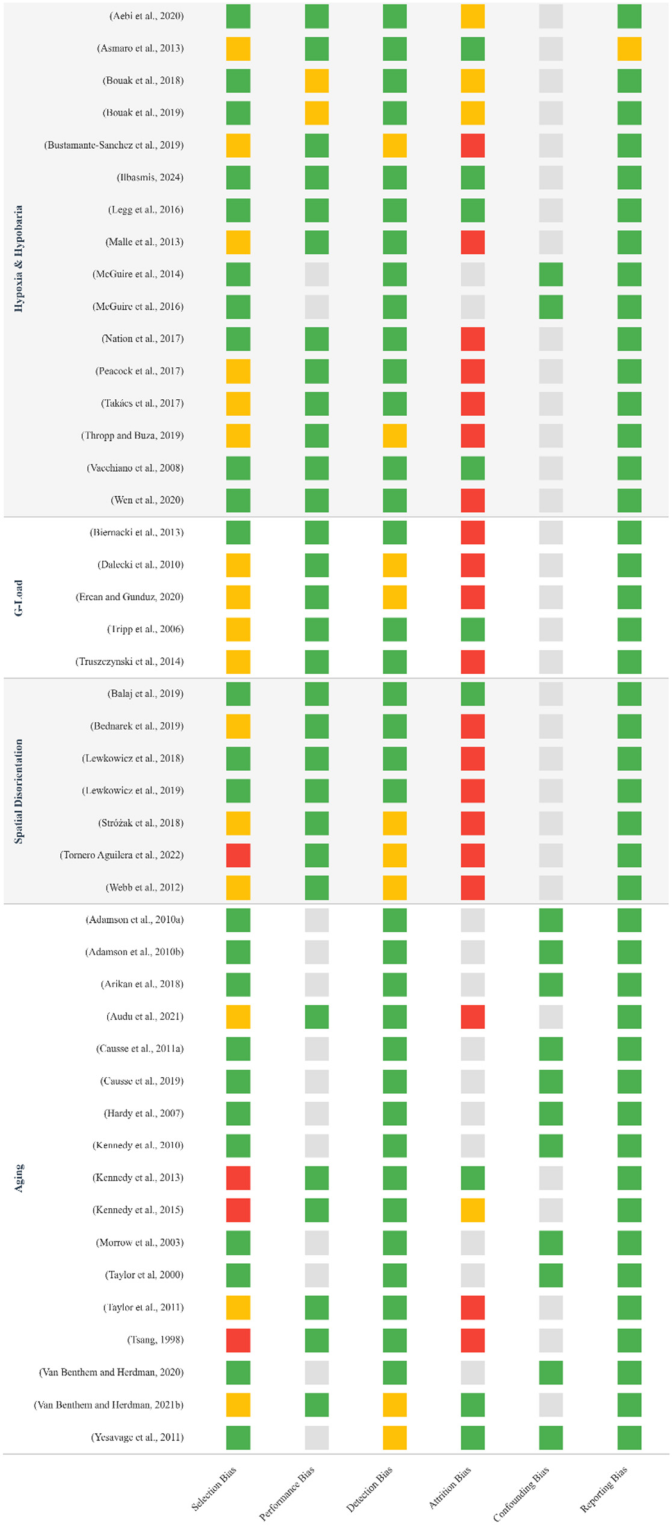

Note. This figure summarizes the risk-of-bias ratings for the 74 studies that exploring the afftect of aviation risk factors on pilot's cognitive modules. Each study was evaluated across six bias domains (selection, performance, detection, attrition, confounding, and reporting) using the Joanna Briggs

Institute (JBI) Critical Appraisal Checklists. Studies are grouped by cognitive module, with colored squares representing the reviewers' judgments (green = low, yellow = moderate, red = high, gray = not applicable). Two independent reviewers assessed methodological quality, and discrepancies were resolved through discussion.
